# Dual‐Crosslinked Betaine‐Based Amphiphilic Hydrogel as a Promising Vitreous Substitute: Anti‐Adhesion, Anti‐Fouling, and Anti‐Cell Proliferation

**DOI:** 10.1002/advs.202413455

**Published:** 2025-06-30

**Authors:** Yuting Cai, Yun Tan, Jian Cao, Xiaoyuan Zhou, Xingyuan Li, Jianghua Li, Chuntai Liu, Yi Zhang, Yun Li

**Affiliations:** ^1^ Department of Ophthalmology Hunan Clinical Research Center of Ophthalmic Disease The Second Xiangya Hospital Central South University Changsha 410011 China; ^2^ Hunan Provincial Key Laboratory of Micro & Nano Materials Interface Science College of Chemistry and Chemical Engineering Central South University Changsha 410083 China; ^3^ State Key Laboratory of Chemistry and Utilization of Carbon‐Based Energy Resources College of Chemistry Xinjiang University Urumqi Xinjiang 830017 China; ^4^ Jiangxi Province Division of National Clinical Research Center for Ocular Diseases Jiangxi Clinical Research Center for Ophthalmic Disease, Jiangxi Research Institute of Ophthalmology and Visual Science Affiliated Eye Hospital of Nanchang University Nanchang 330006 China; ^5^ Key Laboratory of Materials Processing and Old Ministry of Education Zhengzhou University Zhengzhou 50002 China; ^6^ Key Laboratory of Oil and Gas Fine Chemicals of Ministry of Education College of Chemical Engineering Xinjiang University Urumqi Xinjiang 830017 China

**Keywords:** amphiphilic polymer, injectable hydrogels, self‐assembly, vitreoretinopathy, vitreous substitute

## Abstract

Developing an ideal vitreous substitute remains a significant challenge in ophthalmic surgery. This study introduces a novel dual‐crosslinked betaine‐based amphiphilic polymer hydrogel (BAPHs) synthesized via in situ free radical copolymerization. The resulting BAPHs exhibit tunable viscoelasticity, intrinsic self‐healing property, injectability, stable micromorphology, and optical transparency, all modifiable by adjusting the chemical cross‐linker content. When tested in a vitrectomized rabbit model, the BAPHs demonstrated optimal optical properties, mechanical stability, and promising biocompatibility. These results suggest that the BAPHs offer a transformative approach to vitreous substitution, with the potential to overcome the limitations of current clinical materials, offering both structural and functional benefits.

## Graphical abstract

A novel multiple cross‐linked betaine‐type amphiphilic polymer hydrogels are developed via in situ free radical copolymerization. The resultant formation, named P(SBMA‐co‐AANa) hydrogel, functions as a vitreous substitute and demonstrates superior performance in optical properties, support capacity, and biocompatibility on a vitrectomized rabbit model.

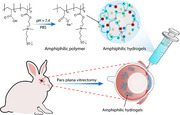

## 1 Introduction

The vitreous body, constituting 80% of the intraocular volume, plays a critical role in ocular homeostasis and various pathological conditions.^[^ ^1^, ^2^ ^]^ Vitreoretinal diseases, which affect ≈8.56% of the global population, are a leading cause of bilateral visual impairment,^[^ ^3^ ^]^ constituting a major threat to global visual health and quality of life.^[^ ^4^, ^5^, ^6^, ^7^ ^]^ Multiple pathologies such as degradation, inflammation, infection, and vasculopathy, often contribute to the onset of vitreoretinal disorders,^[^ ^8^, ^9^ ^]^ which would lead to a surgical intervention known as pars‐plana vitrectomy (removal and substitution of the vitreous body).^[^ ^10^, ^11^ ^]^ However, current vitreous substitutes, including silicone oils (SO),^[^ ^12^, ^13^ ^]^ gases,^[^ ^14^ ^]^ and balanced salt solutions (BSS)^[^ ^15^ ^]^ exhibit both short‐ and long‐term limitations, such as insufficient support, uncomfortable postoperative posture requirements, cataract and glaucoma induction, and unsatisfactory biocompatibility.^[^ ^16^ ^]^ In brief, the development of innovative materials for long‐term endo‐tamponade substitutes is therefore crucial, urging rapid and innovative advancements in this field.

An ideal vitreous substitute simulates the natural vitreous body's key properties, including transparency, biocompatibility, osmotic pressure, elasticity, and buffer capacity, while avoiding limitations such as liquefaction, age‐related biodegradation, and uncontrolled cellular proliferation.^[^ ^17^, ^18^ ^]^ Furthermore, it should demonstrate surgical feasibility, facilitate introduction through diminutive incisions, and exhibit anti‐inflammatory and anti‐infection properties.^[^ ^19^, ^20^ ^]^ Polymer‐based hydrogels have emerged as promising vitreous substitutes due to their exceptional biocompatibility, heightened aqueous content, optical clarity, and viscoelasticity.^[^ ^20^, ^21^, ^22^, ^23^, ^24^ ^]^ Natural polymer hydrogels, such as collagen, gelatin, and hyaluronic acid, have been explored for vitreous replacement but suffer from rapid degradation and limited resilience.^[^ ^25^, ^26^ ^]^ Synthetic alternatives, such as polyvinyl alcohol,^[^ ^27^, ^28^ ^]^ polyethylene glycol,^[^ ^20^, ^25^, ^29^ ^]^ and polyacrylamide,^[^ ^30^ ^]^ have also been investigated for vitreous substitution,^[^ ^31^, ^32^ ^]^ though challenges related to in vivo durability and degradation remain.^[^ ^33^, ^34^, ^35^ ^]^

Despite significant advancements in self‐crosslinked polymeric vitreous substitutes,^[^ ^19^, ^36^ ^]^ achieving long‐term stability while minimizing degradation and controlling network inhomogeneity continues to pose major challenges.^[^ ^37^ ^]^ The random construction of a 3D network results in spatial inhomogeneity, creating opportunities for cell penetration and protein adhesion.^[^ ^34^ ^]^ Strategies to improve mechanical durability, adaptive viscoelasticity, and osmotic regulation, while avoiding adverse reactions like inflammation, remain crucial for developing viable long‐term vitreous substitutes.^[^ ^38^ ^]^ A long‐term vitreous substitute must, on one hand, possess high transparency, a stable swelling rate, biological inertness, resistance to contamination, and tolerance to microenvironment changes.^[^ ^39^ ^]^ On the other hand, it should also exhibit a self‐adaptive and tunable structure to prevent breakage during injection.^[^ ^40^ ^]^ Achieving this balance requires either serendipitous discovery or meticulous design.

**Figure 1:**
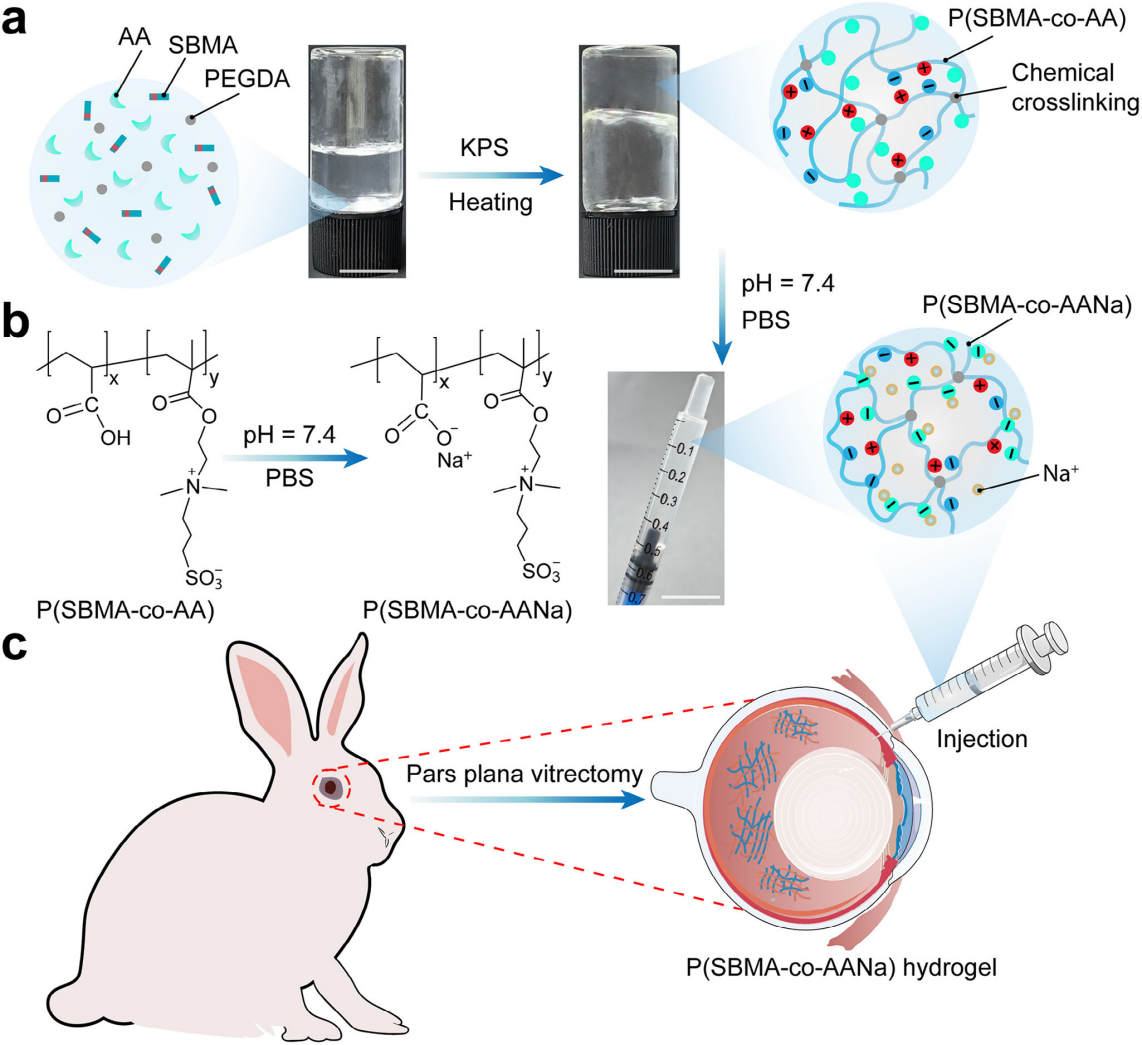
Schematic diagram of P(SBMA‐co‐AANa) hydrogel's chemical structure and injection experiments. a) Schematic diagram of P(SBMA‐co‐AANa) hydrogel preparation process. b) Chemical structure of P(SBMA‐co‐AANa) hydrogel. c) P(SBMA‐co‐AANa) as a vitreous substitute hydrogel through a small gauge needle injecting into rabbit eyes. Scale bar = 1 cm.

In this study, we introduce an innovative approach to the creation of an injectable BAPHs vitreous substitute, synthesized using small amounts of chemically cross‐linked, electrostatic, and hydrogen‐bond cross‐linked poly (betaine methacrylate sulfonate‐co‐acrylic acid) P(SBMA‐co‐AANa) via in situ radical copolymerization (**Figure** **1**a,b). The incorporation of minute quantities of chemical cross‐linkages imparts the hydrogel with the requisite mechanical robustness to withstand shear forces, thereby preserving the structural integrity of its 3D lattice. The hydrogel's self‐adaptive and self‐repair properties, enabled by hydrogen bonding and electrostatic cross‐linking between quaternary ammonium and sulfonic groups, enable it to recover from shear‐thinning after injection.^[^ ^41^ ^]^ Moreover, the presence of quaternary ammonium groups enhances its antibacterial properties, reducing the risk of inflammatory responses post‐implantation. The carboxyl and carboxylate groups inherent to the hydrogel serve as efficacious pH buffers,^[^ ^42^, ^43^ ^]^ enabling the hydrogel to tolerate fluctuations in the acid‐base environment of the vitreous body,^[^ ^9^, ^44^ ^]^ thereby safeguarding against degradation and ensuring long‐term physicochemical stability. To evaluate the efficacy and biocompatibility of P(SBMA‐co‐AANa) hydrogels as a potential vitreous substitute, we performed a series of in vivo experiments using a rabbit model (Figure 1c). After injection into the vitreous cavity, the P(SBMA‐co‐AANa) hydrogels exhibited exceptional transparency, robust support capacity, pollutant resistance, and excellent biocompatibility over a 90‐day period. These findings suggest that this amphiphilic injectable hydrogel holds great promise as a vitreous substitute, serving both its biological function and maintaining high transparency and long‐term stability. We anticipate that this work will serve as a valuable reference for future clinical research and the development of advanced vitreous body substitutes.

## 2 Results and Discussion

### 2.1 Microstructure of P(SBMA‐co‐AANa) Hydrogels

**Figure 2:**
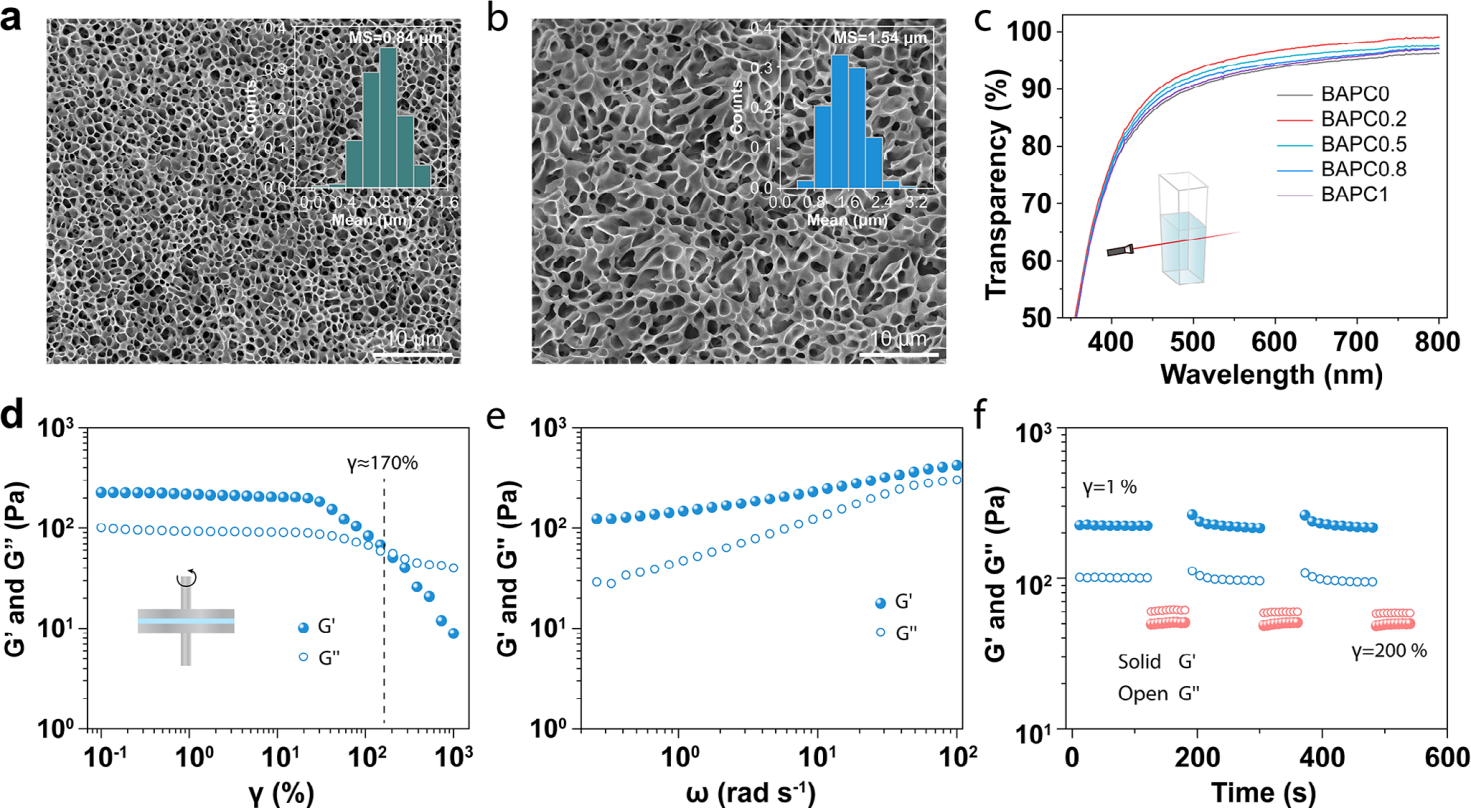
Microstructure and characterization of P(SBMA‐co‐AANa) hydrogels. The SEM images of a) BAPC1 and b) BAPC0.8, scale bar = 10 µm. The illustrations were the statistical analysis of the pore size of the corresponding hydrogels. c) The transparency of hydrogel in the visible light region. d) The oscillation strain scanning of BAPC0.8. e) The frequency scanning of BAPC0.8. f) The changes of *G*' and *G*“” of BAPC0.8 under low (1%) and high (200%) oscillatory shear strain.

To investigate the influence of cross‐linker content on the microstructure of hydrogels, the scanning electron microscopy (SEM) tests of P(SBMA‐co‐AANa) hydrogels were performed (**Figure** **2**a,b). The SEM images of P(SBMA‐co‐AANa) hydrogels (Based on the different content of the chemical cross‐linker, P(SBMA‐co‐AANa) hydrogels are defined as BAPCx, where x represents the mass concentration (wt.‰) of the chemical cross‐linker relative to the monomer.) displayed a uniform honeycomb‐like 3D network structure. Furthermore, the average pore diameter of BAPC0.8 was ≈1.54 µm (Figure 2b). As the cross‐linker content of BAPC1 was 25% higher than that of BAPC0.8, the average pore size of BAPC1 was reduced to ≈0.84 µm, nearly half the size of that in BAPC0.8 (Figure 2a). This uniform honeycomb network indicates a stable cross‐linking effect between polymer chains, which contributes to favorable viscoelastic properties. However, the excessive cross‐linking degree in BAPC1 compromised its self‐healing and injectability, making it unsuitable as a vitreous substitute. BAPC0.2 and BAPC0.5 also exhibited obvious pore structures, but these pores were mainly caused by ice crystal sublimation during the freeze‐drying process (Figure S1, Supporting Information), and their sizes ranged from 5 to 10 µm. This further indicates that the degree of cross‐linking in BAPC0.2 and BAPC0.5 was too low to impart them with viscoelasticity comparable to BAPC0.8.

### 2.2 Physicochemical Properties of the Hydrogel

As it serves as the primary medium for light entering the eye, the transparency of the vitreous substitute is a critical physicochemical property of interest. In our system, the amphiphilic polymer (BAPC0) maintains a transparency of ≈80%–95% across the visible light spectrum (wavelengths of 400–800 nm), with transparency exceeding 90% beyond 500 nm (Figure 2c). This phenomenon is attributed to the multiple physical interactions within the amphiphilic polymer, particularly ionic bonding and polymer entanglement. These interactions lead to localized cross‐linking irregularities and microphase separation, which can reduce the hydrogel's transparency. Interestingly, the reduction in transparency at the lower wavelengths (400‐460 nm) may have a beneficial “blue light blocking” effect, potentially mitigating blue light‐induced retinal photodamage, especially to the macula.^[^ ^45^, ^46^, ^47^ ^]^ By adjusting the chemical cross‐linker content, the pore size of the hydrogel network can be modified, influencing transparency. For example, betaine‐based hydrogels with chemical cross‐linkers exhibit higher transparency compared to BAPC0 due to a more fixed cross‐linked network. However, as the cross‐linker content increases, the hydrogel's transparency decreases due to the denser polymer network. Therefore, optimizing both physical and chemical cross‐linking components can enhance transparency without compromising other key properties of the hydrogel.

Besides, an appropriate refractive index and density can reduce the likelihood of postoperative anisometropia and decrease the need for specific postoperative positioning. Evaluation results of the refractive index and density of the betaine‐based hydrogel showed that, compared to refractive index of SO (1.404), the refractive index of the betaine‐based hydrogel (1.341‐1.390) is closer to that of the natural vitreous body (1.3345‐1.3348),^[^ ^9^ ^]^ effectively preventing postoperative hyperopia caused by SO (Figure S2, Supporting Information). Additionally, the density of the hydrogel is slightly higher than that of water, allowing it to press against the retina through gravitational settling, thereby eliminating the need for patients to maintain a prone position for extended periods post‐surgery (Figure S3, Supporting Information).

The ideal vitreous substitute also be able to be injected through small incisions and remain stable in the eye. Therefore, the chemical cross‐linking content of BAPHs was adjusted to make them have good injectable property, self‐healing ability, and elasticity similar to natural vitreous body (storage modulus: 10^2^–10^5^ Pa), and have the characteristics of slow degradation.^[^ ^34^, ^38^ ^]^ The viscosity assessment of rabbit vitreous and BAPC0.8 showed that the viscosity of the hydrogel is slightly higher than the viscosity of rabbit vitreous, but both exhibit significant shear‐thinning behavior (Figure S4, Supporting Information). Based on rheology amplitude scanning of hydrogel, we confirmed that the viscoelasticity and self‐healing property of hydrogel were the most appropriate as a substitute for the vitreous body when the content of chemical cross‐linker (poly (ethylene glycol) diacrylate, PEGDA‐1000) was 0.8 wt.‰ (Figure 2d). The storage modulus (*G*') and loss modulus (*G*“”) of BAPC0.8 hardly changed with the increase of shear strain when shear strain (γ) was less than 30%, and always maintained the gel‐state (*G*“ > *G*”“). Additionally, when the γ was ≈170%, the *G*” and *G*“” of the BAPC0.8 were equal, indicating that the hydrogel possesses self‐healing and injectable properties. However, when PEGDA‐1000 was increased to 0.1%, BAPC1 had good viscoelasticity and wide linear viscoelastic region but maintained a *G*' far greater than the *G*“” (the presence of γ < 1000%). These results indicate that the hydrogel is mainly chemically cross‐linked and lacks self‐healing and injectable properties, so it is not suitable as an injectable vitreous substitute (Figure S5, Supporting Information). Compared to BAPC1, BAPC0.8 exhibits suitable viscoelasticity and self‐healing properties as a vitreous substitute. The reason lies in the introduction of a small amount of cross‐linker, which not only enhances the viscoelasticity of the amphiphilic polymer hydrogel, but also leverages multiple physical cross‐linking interactions to impart self‐healing and injectability.

The addition of a small amount cross‐linker enhanced the viscoelasticity of multiple cross‐linking hydrophilic polymer hydrogels and retained the self‐healing and injectable properties. The *G*' of the BAPC0.8 was always greater than *G*“” in the whole frequency range (*ω* = 0.01–100 rad s^−1^), and the *G*' of BAPC0.8 varied from 100 to 500 Pa, which was in line with the mechanical strength of natural vitreous body (Figure 2e). In addition, in the ω = 0.01–100 rad s^−1^ frequency range, the *G*' of BAPC1 was always greater than the *G*“”, which shows the viscoelasticity of hydrogel (Figure S6, Supporting Information). However, due to its main manifestation of chemical cross‐linking, weak injectability, and self‐healing properties, it was not suitable as an injectable vitreous body substitute. Moreover, BAPC0.2 and BAPC0.5 also cannot be used as injectable vitreous substitutes because of their low degree of cross‐linking and their viscous flow characteristics in the ω<0.5 rad/s frequency range (Figure S7, Supporting Information). The self‐healing properties of BAPC0.8 were further investigated by analyzing the relationship between *G*' and *G*“” under low shear strain (γ = 10%) and high shear strain (γ = 200%) at 1 Hz (Figure 2f). The *G*' was always much greater than the *G*“” under the shear strain of the γ = 10%, and both the *G*' and *G*“” remained invariant with time, implying the solid‐like and inherent elasticity of the multiple cross‐linked hydrogels. When the shear strain was increased to 200%, an overturn of the *G*' and *G*“” (*G*“<*G*”') suggested the disruption of the hydrogen‐bonded and electrostatic interaction cross‐linking. After three cycles of low and high shear strain, the *G*' and *G*“” of the BAPC0.8 had good reproducibility, further indicating that hydrogen bonding and electrostatic interaction in BAPC0.8 would be quickly reconstructed and dissociated to achieve self‐healing and injectability. Therefore, BAPC0.8 was selected as a suitable vitreous substitute for further experiments.

### 2.3 In Vitro Biocompatibility Test

**Figure 3:**
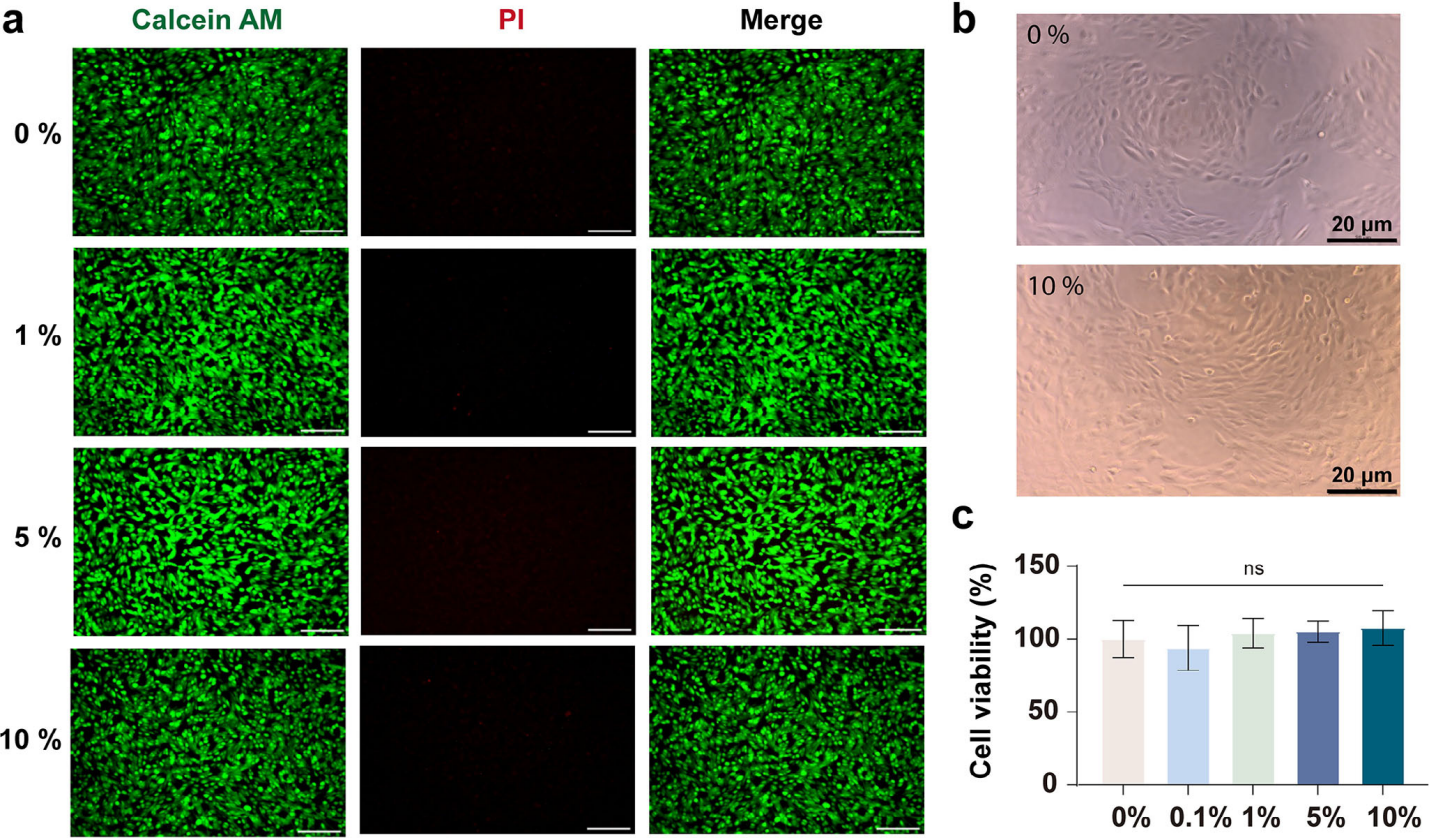
Cytotoxicity of P(SBMA‐co‐AANa) hydrogel. a) Live/Dead staining images of ARPE‐19 cells treated with different concentrations of BAPC0.8 for 24 h, scale bar = 100 µm. b) Microscopic images of ARPE‐19 cells after 24 h of gel treatment, scale bar = 20 µm. c) Cell viability was measured after 24 h of co‐incubation with the gels (0%–10%), and there was no statistical difference among the groups. The statistical analysis in c was performed using one‐way ANOVA with Dunnett's post hoc test, and data in c are presented as means ± standard deviation (SD), n = 6.

Given the suitable physiological and chemical properties of P(SBMA‐co‐AANa) hydrogel, its cytotoxicity was further assessed using the ARPE‐19 cell line via Live/Dead Cell Staining (**Figure** **3**a) and CCK‐8 assay (Figure 3b). After co‐culturing ARPE‐19 cells with BAPC0.8 for 24 h, no significant differences were observed in the number of viable ARPE‐19 cells (green‐stained) across all experimental groups, with cell viability nearing 100% (Figure 3c and *P* > 0.05). These results suggest that the hydrogel is non‐cytotoxic within the tested concentration range, supporting its potential for further in vivo evaluation.

### 2.4 ARPE‐19 Cells Migration Inhibition Assay

**Figure 4:**
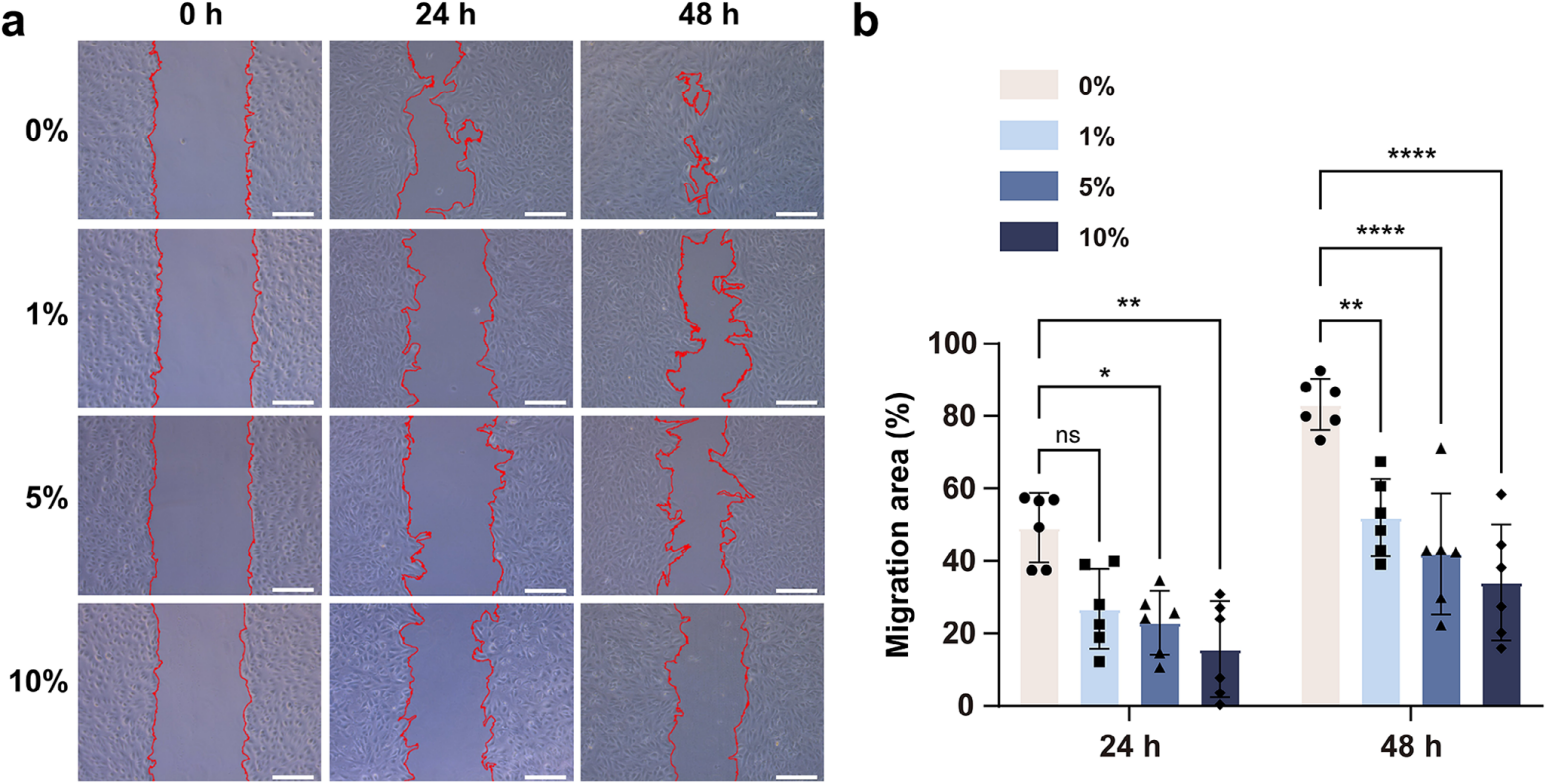
ARPE‐19 cells migration inhibition assay of P(SBMA‐co‐AANa) hydrogel. a) Representative migration images of ARPE‐19 cells treated with different concentrations of BAPC0.8 for 0, 24, and 48 h, scale bar = 20 µm. b) Statistical diagram of migration area. Data were presented as means ± SD. Statistical significance at 24 h was calculated by Kruskal‐Wallis test with Dunn's multiple comparisons test, and 48 h was calculated by one‐way ANOVA with Dunnett's post hoc test. n = 6. *P <  0.05, **P < 0.01, ****P < 0.0001.

Then, we explored the effects of P(SBMA‐co‐AANa) hydrogel on the migration of ARPE‐19 cells, a key process in proliferative vitreoretinopathy (PVR).^[^ ^48^ ^]^ PVR is a common cause of failed vitrectomy,^[^ ^49^, ^50^ ^]^ characterized by the activation and migration of retinal pigment epithelial cells, leading to proliferation, epithelial‐mesenchymal transition, and collagen formation.^[^ ^51^, ^52^ ^]^ **Figure** **4** demonstrates that even a low concentration of BAPC0.8 effectively inhibited ARPE‐19 cell migration in vitro, highlighting its potential for preventing PVR.

### 2.5 In Vitro Anti‐Cell and Anti‐Protein Adhesion Test

**Figure 5:**
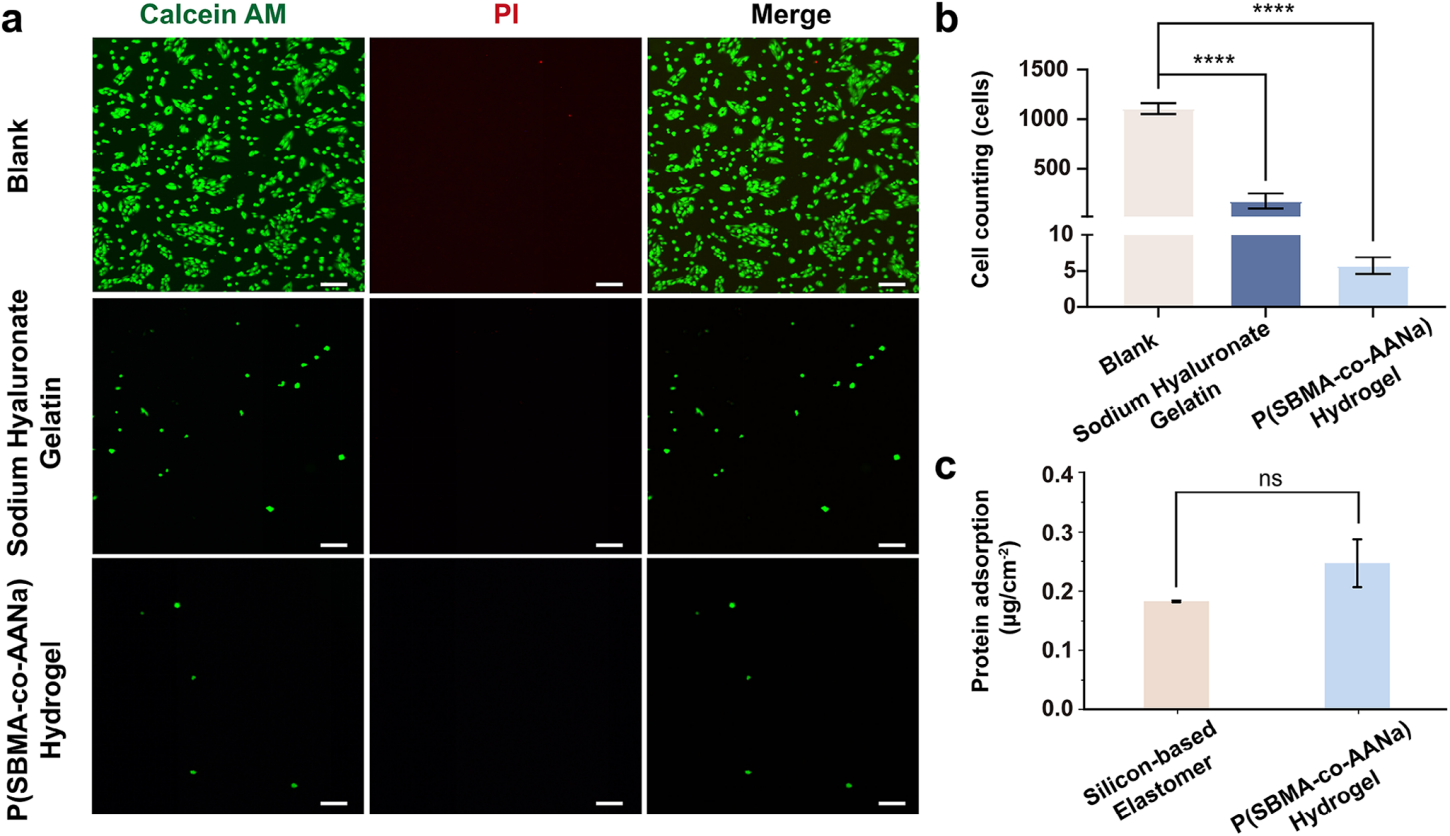
Anti‐cell and anti‐protein adhesion test of P(SBMA‐co‐AANa) hydrogel. a) Live/Dead staining images of ARPE‐19 cells seeded into the blank bottom of 24‐well plates, the surface of sodium hyaluronate, and P(SBMA‐co‐AANa) hydrogel for 24 hours, scale bar = 100 µm. b) Cartogram of anti‐cell adhesion showed that compared with the control group, P(SBMA‐co‐AANa) hydrogel had better anti‐cell adhesion properties, statistical significance was calculated by one‐way ANOVA with Dunnett's post hoc test. n = 3, ****P < 0.0001. c) Protein adsorption of P(SBMA‐co‐AANa) hydrogel, statistical significance was calculated by unpaired t‐test. n = 3. Data in b and c were presented as means ± SD.

Another critical property of vitreous substitutes is to maintaining long‐term transparency, which requires the material to exhibit low immunogenicity. To detect the anti‐fouling properties of P(SBMA‐co‐AANa) hydrogel, we performed the anti‐cell and anti‐protein adhesion experiments. As shown in **Figure** **5**a,b, ARPE‐19 cells were seeded on 24‐well plates coated with sodium hyaluronate gelatin and P(SBMA‐co‐AANa) hydrogel. After 1 day of cultivation and removal of non‐adherent cells, the adhesion of ARPE‐19 cells to P(SBMA‐co‐AANa) hydrogel and sodium hyaluronate gelatin decreased significantly. In contrast, a substantial amount of green fluorescence was observed in the blank control group, indicating high levels of cell adhesion. In addition, compared to silicon‐based elastomers, which exhibit excellent anti‐fouling properties (0.18 µg cm^−2^), the nonspecific protein adsorption capacity of the P(SBMA‐co‐AANa) hydrogels was found to be ≈0.24 µg cm^−2^, with no significant difference (Figure 5c). These findings confirm that the introduction of betaine‐based hydrogel provides exceptional anti‐fouling properties in vitreous substitutes.

### 2.6 In Vivo Biocompatibility of P(SBMA‐co‐AANa) Hydrogel

**Figure 6:**
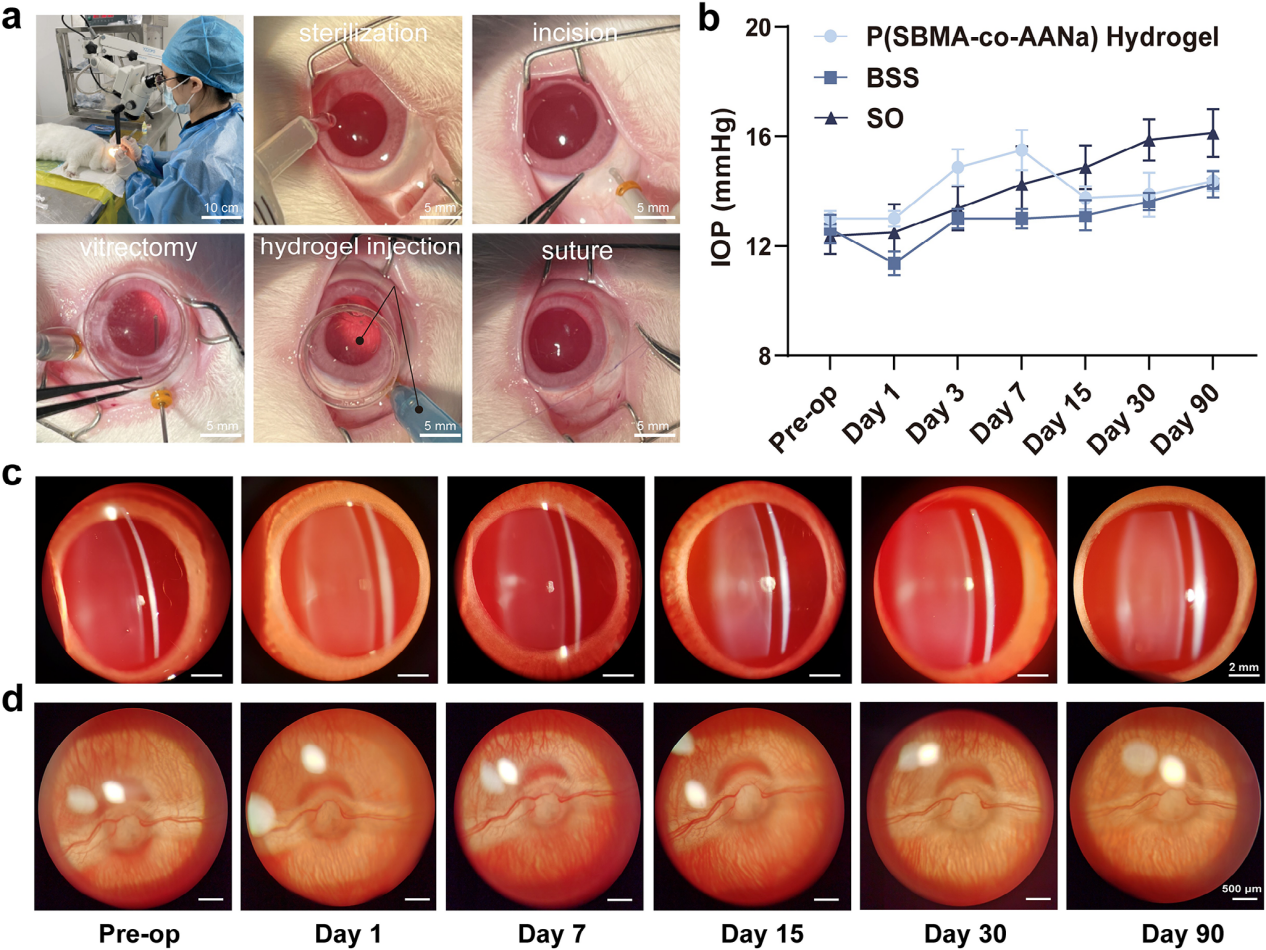
Ocular biocompatibility after hydrogel endotamponade. a) Standard vitrectomy was performed by an experienced surgeon in the sterilized operation room during which the P(SBMA‐co‐AANa) hydrogel was injected into the vitrectomized eye. b) Intraocular pressure measurement showed a transient rise from day 3 to day 7 (yet within a normal range), and back to baseline at day 15. Photography of the anterior segment c) and fundus d) on days 0 (pre‐operation), 1, 7, 15, 30, and 90, showed perfectly quiet eye, proving the substitute's excellent biocompatibility. The IOP data were presented as mean ± SD, and calculated by one‐way ANOVA with Dunnett's post hoc test, n = 4. Pre‐op: pre‐operation.

To evaluate the in vivo biocompatibility, P(SBMA‐co‐AANa) hydrogel was first injected into the subcutaneous and subconjunctival spaces of rabbits. As shown in Figure S8 (Supporting Information), the rabbit fur grew normally, no signs of skin irritation, or conjunctival hyperemia/edema was observed within the first month after injection. Subsequently, the hydrogel was injected into the vitreous cavity of rabbits following standard pars plana vitrectomy (**Figure** **6**a; Figure S9 and Video S1, Supporting Information), with comparisons made to BSS and SO. During the observation period, the rabbits maintained a normal diet and circadian rhythm, and both anatomical and functional evaluations of intraocular reactions were conducted. As shown in Figure 6b, following hydrogel endotamponade, a transient, mild increase in intraocular pressure (IOP) was observed, but it remained within the normal range (11–21 mmHg) and returned to baseline by day 15, with no significant differences between the BSS and SO groups by day 90. The slit‐lamp examinations also showed clear visibility of fundus vessels in all three groups, with no signs of congestion, anterior chamber flare, vitreous haze, or other inflammatory reactions throughout the observation period (Figure 6c,d; Figures S10 and S11, Supporting Information), indicating that the hydrogel did not induce obvious inflammation or swelling.

### 2.7 Anatomical and Functional Evaluation of Hydrogel‐Injected Eye

**Figure 7:**
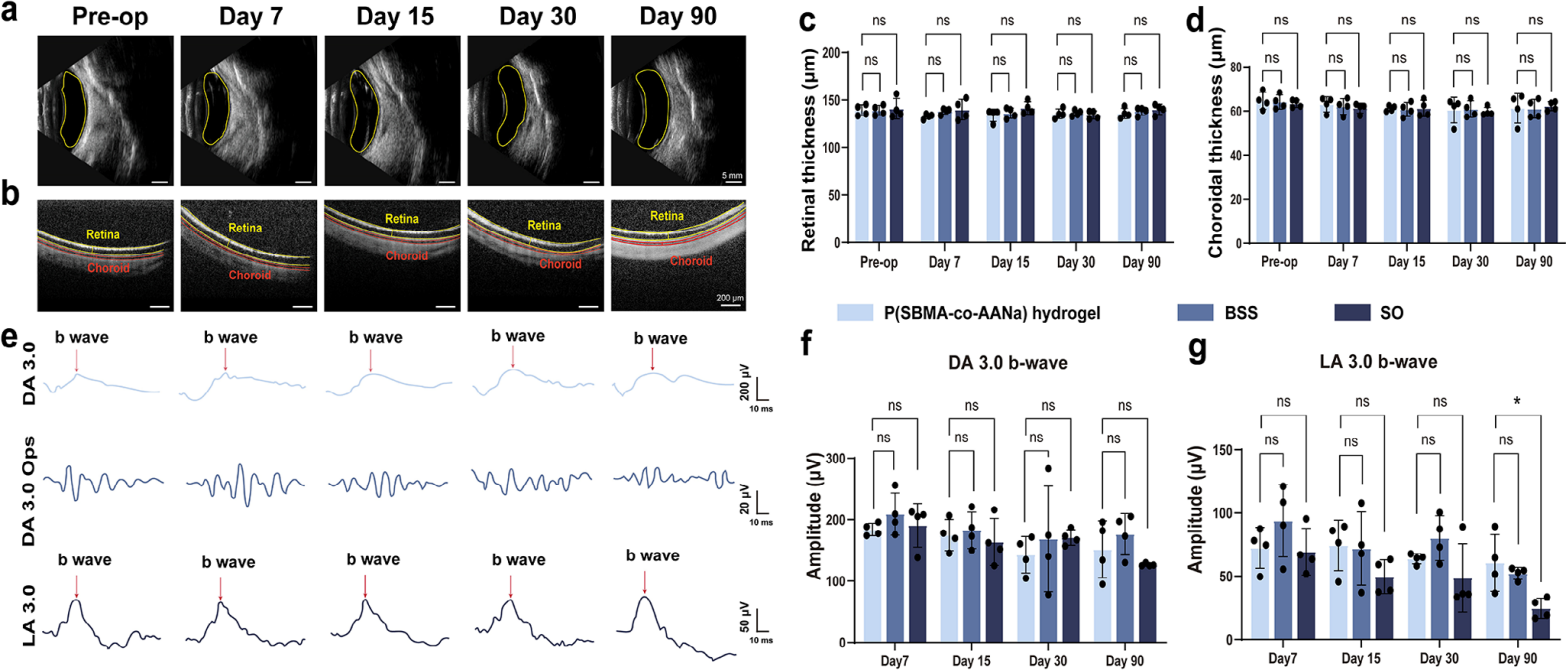
Anatomical and functional results after the hydrogel endotamponade. a) B‐ultrasonography of the hydrogel‐injected eye on days 0, 7, 15, 30, and 90 showed no obvious difference in the turbidity degree of the vitreous cavity. b) Optic coherent topography images showed a similar structural status of the retina at each time point, while retinal thickness c) and choroidal thickness d) measurements were unchanged up to day 90. e) Dark‐adapted (DA) and light‐adapted (LA) ERG showed no obvious difference between the BSS group and the hydrogel group throughout the observation, revealing normally functioned retinae after hydrogel endotamponade. f,g) Amplitude statistics of ERG. The retinal and choroidal thicknesses were presented as mean ± SD and were analyzed using one‐way ANOVA with Dunnett's post hoc test, n = 4. The ERG wave amplitude data were also analyzed by one‐way ANOVA with Dunnett's post hoc test. Since the DA 3.0 Ops amplitude data on day 7 and the LA 3.0 b‐wave amplitude data on day 30 deviated from a normal distribution, the Kruskal‐Wallis test with Dunn's multiple comparisons test was employed for analysis. n = 4, *P < 0.05.

SO, the most commonly used long‐term endotamponade, often appears as hyperechoic spots when injected into the vitreous cavity, as seen in B‐ultrasonography (Figure S11, Supporting Information), potentially complicating clinical assessments. In contrast, B‐ultrasonography of hydrogel‐injected eyes on days 0, 7, 15, 30, and 90 showed clear vitreous cavities, with no signs of inflammation or retinal detachment (**Figure** **7**a). The gray value measurements of the vitreous cavity indicated that the P(SBMA‐co‐AANa) hydrogel outperformed the SO group, as the SO group exhibited persistently higher gray value, statistically different from the other two groups by day 30 (Figures S12 and S13, Supporting Information). Besides, SO restricts substance exchange, potentially leading to intraocular metabolic imbalances, accelerated cataract formation, and challenges in drug delivery. While the fibrous network structure of hydrogels facilitates substance exchange, thereby supporting the homeostasis of the intraocular microenvironment and providing a robust foundation for future drug loading and release (Figure S14, Supporting Information). Real‐time spectral‐domain optical coherence tomography images showed similar retinal structure in both the hydrogel and control groups at all time points (Figure 7b), with no significant changes in retinal and choroidal thickness up to day 90 (Figure 7c,d; Figure S15, Supporting Information). Optical coherence tomography angiography also indicated that the gel had no negative effect on retinal microcirculation within 90 days (Figure S16, Supporting Information). More importantly, the highly cross‐linking structure of P(SBMA‐co‐AANa) hydrogel remained detectable on day 30 post‐surgery, indicating its potential as a vitreous substitute capable of providing at least 30 days of support necessary for retinal self‐repair (Video S2, Supporting Information). Electroretinogram (ERG) results showed no obvious differences between the hydrogel‐injected (Figure 7e–g; Figure S17, Supporting Information) and BSS‐injected eyes (Figure S18, Supporting Information) at each time point, indicating that the postoperative visual function of hydrogel‐injected group remained normal. However, with the extension of the observation period, the LA 3.0 amplitudes in the SO group gradually declined and showed a statistically significant difference by Day 90, which is consistent with previous findings that long‐term silicone oil tamponade may lead to ERG amplitude reduction.^[^ ^53^ ^]^ Furthermore, histopathological sections on day 30 revealed that the cardiac, hepatic, renal, and retinal structures of the hydrogel‐injected rabbits remained intact (Figure S20, Supporting Information). The inflammatory cytokine levels in the hydrogel‐injected eyes were also not significantly different from those in the SO group (Figure S21, Supporting Information). All groups exhibited steady weight gain throughout the observation period (Figure S22, Supporting Information), revealing a preliminary good systemic safety of P(SBMA‐co‐AANa) hydrogel.

Although the present study provides promising results, there are several limitations that should be addressed in future work. First, future studies should use pigmented animal models to better simulate retinal characteristics in different humans’ races. Second, the observation period was relatively short in this study, degradation of the hydrogel in vivo should be assessed in longer term. Lastly, the transparency of the hydrogel was found to be between 80–90%, which may not be clear enough for patients with high visual expectation shortly after surgery, but may improve over time as the hydrogel degrades. However, the hydrogel specifically filters blue light in the 400–500 nm range, which may help reduce the risk of blue light‐induced damage to the retina and macula.^[^ ^45^, ^46^, ^47^ ^]^ Nonetheless, the P(SBMA‐co‐AANa) hydrogel demonstrates significant potential for ocular applications, showing excellent anatomical and functional tolerance.

## 3 Conclusion

This study introduces an innovative vitreous substitute developed through the integration of multiple cross‐linked betaine‐type amphiphilic polymers via in situ free radical copolymerization. The resulting amphiphilic polymer hydrogels exhibit tunable viscoelasticity, self‐healing properties, injectability, stable micromorphology, and targeted transparency. Notably, when utilized as a vitreous substitute, the hydrogel demonstrates remarkable biocompatibility and powerful anti‐migration/proliferation capabilities, both of which are essential for surgical success for vitrectomy. These findings not only highlight the potential of this hydrogel as a promising vitreous replacement but also offer a robust framework for the future development of hydrogel‐based vitreous substitutes, positioning this approach as a significant advancement in the field of artificial vitreous bodies.

## 4 Experimental Section

Materials and methods are provided in Supporting Information.

## Conflict of Interest

The authors declare no conflict of interest.

## Author Contributions

Y.C. and Y.T. contributed equally to this work. Y.C., Y.T., J.C., Y.Z., and Y.L. conceived and designed experiments. Y.C., Y.T., J.C., X.Z., X.L., and J.L. performed the surgeries and the in vitro and in vivo experiments. Y.C. and Y.T. analyzed and interpreted data. Y.C., Y.T., J.C., C.L., Y.Z., and Y.L. wrote and revised the manuscript. Y.T., Y.Z., and Y.L. provided administrative and material support. Y.Z. and Y.L. supervised and coordinated all aspects of the work.

## Supporting information



Supporting Information.

Supplemental Video 1

Supplemental Video 2

## Data Availability

The data that support the findings of this study are available from the corresponding author upon reasonable request.
