# Supplementary material for: Dual‐Crosslinked Betaine‐Based Amphiphilic Hydrogel as a Promising Vitreous Substitute: Anti‐Adhesion, Anti‐Fouling, and Anti‐Cell Proliferation
Source: Adv Sci (Weinh). 2025 Jun 30;12(36):e13455. doi: 10.1002/advs.202413455 (PMC12462983; doi:10.1002/advs.202413455)
Supplement: Supplementary file 1 — Supporting Information. [file ADVS-12-e13455-s001.docx]

Supporting Information

**Dual-Crosslinked** **Betaine-Based Amphiphilic Hydrogel as a Promising Vitreous Substitute: Anti-Adhesion, Anti-Fouling, and Anti-Cell Proliferation**

*Yuting Cai, Yun Tan, Jian Cao, Xiaoyuan Zhou, Xingyuan Li, Jianghua Li, Chuntai Liu, Yi Zhang,* Yun Li**

**Table of Content**

**Figure S1.** The SEM images of freeze-dried BAPHs. 1

**Figure S2**. The refractive index of BAPHs and SO. 1

**Figure S3.** The density of BAPHs. 1

**Figure S4.** The relationship between the viscosity of the BAPC0.8 and rabbit vitreous with shear rate. 2

**Figure S5.** Rheological characteristics with the oscillation strain scanning of BAPC1. 2

**Figure S6.** Rheological characteristics with the frequency scanning of BAPC1. 3

**Figure S7.** Rheological characteristics with the frequency scanning of weakly crosslinked P(SBMA-co-AANa) hydrogel. 3

**Figure S8.** Subcutaneous and subconjunctival injection safety testing. 4

**Figure S9.** The procedure of vitrectomy and hydrogel injection. 4

**Figure S10.** The anterior segment, fundus, and B-ultrasound results of the BSS group. 5

**Figure S11.** The anterior segment, fundus, and B-ultrasound results of the SO group. 5

**Figure S12.** Measuring method of B-ultrasound gray value of vitreous cavity. 6

**Figure S13.** Measuring results of vitreous cavity gray value, indicating no significant difference between the P(SBMA-co-AANa) hydrogel and BSS groups. 6

**Figure S14.** Evaluation of the absorption and release of fluorescein sodium dye by BAPC0.8 P(SBMA-co-AANa) hydrogel and silicone oil. 7

**Figure S15.** Measuring method of retinal and choroidal thickness. 7

**Figure S16**. The OCTA results for the P(SBMA-co-AANa) hydrogel, BSS, and SO groups on days 15, 30 and 90. 8

**Figure S17.** Results of DA 0.01 and LA 3.0 flicker in P(SBMA-co-AANa) hydrogel group. 9

**Figure S18.** The OCT and ERG results of the BSS group. 9

**Figure S19.** The OCT and ERG results of the SO group. 10

**Figure S20.** HE staining results of the retina, heart, liver, and kidney on the 30th day after surgery. 11

**Figure S21.** The interleukin concentration in aqueous humor on day 30. 11

**Figure S22.** The weight gain curves of the three groups. 12


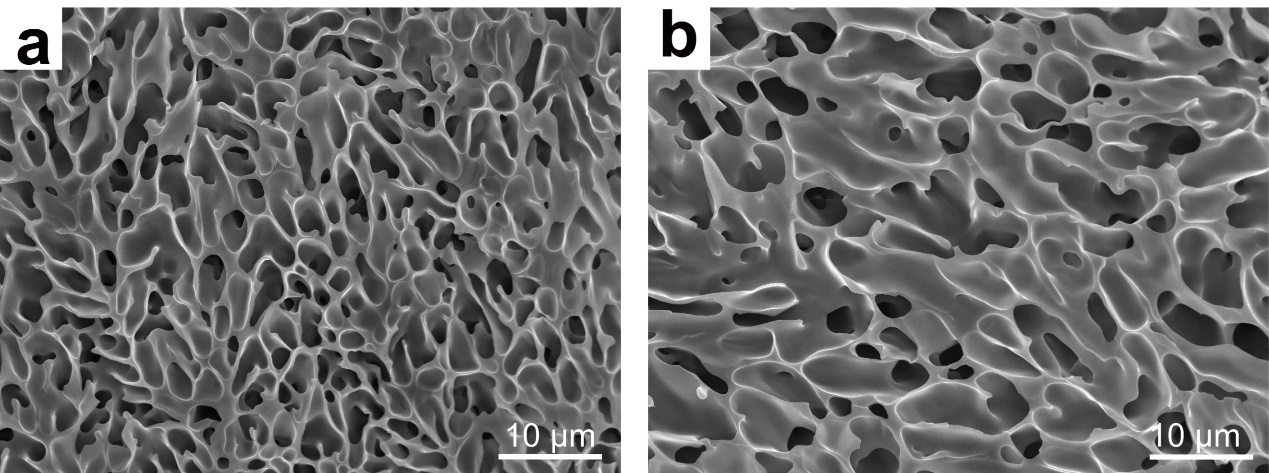


**Figure S1****.** The SEM images of freeze-dried BAPHs. (a) BAPC0.5 and (b) BAPC0.2 (scale bar = 10 μm).


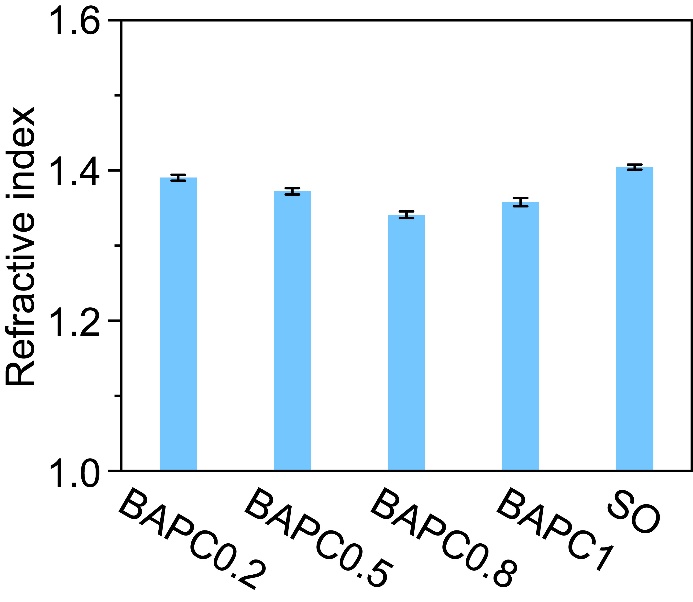


**Figure S2.** The refractive index of BAPHs and SO.


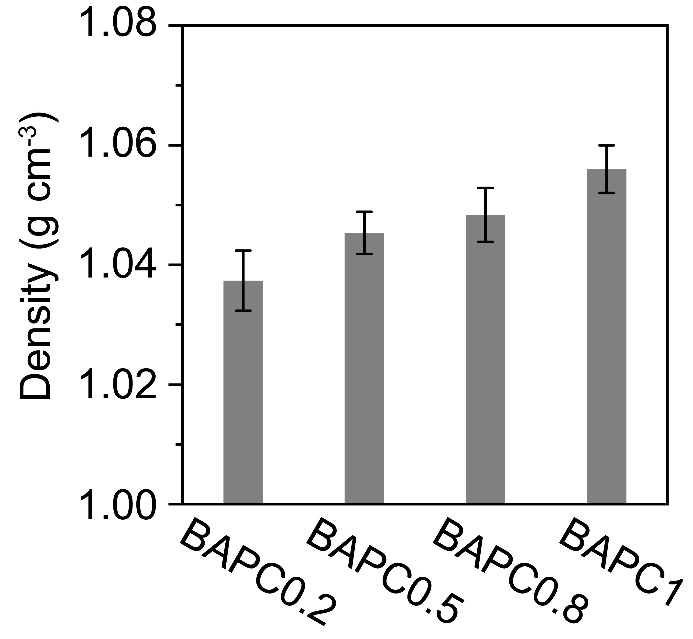


**Figure S3.** The density of BAPHs.


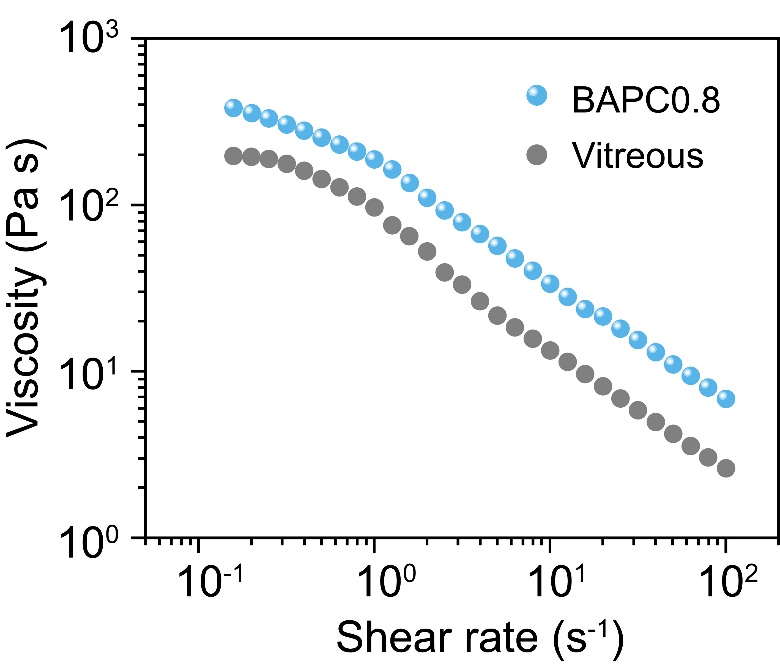


**Figure S4.** The relationship between the viscosity of the BAPC0.8 and rabbit vitreous with shear rate.


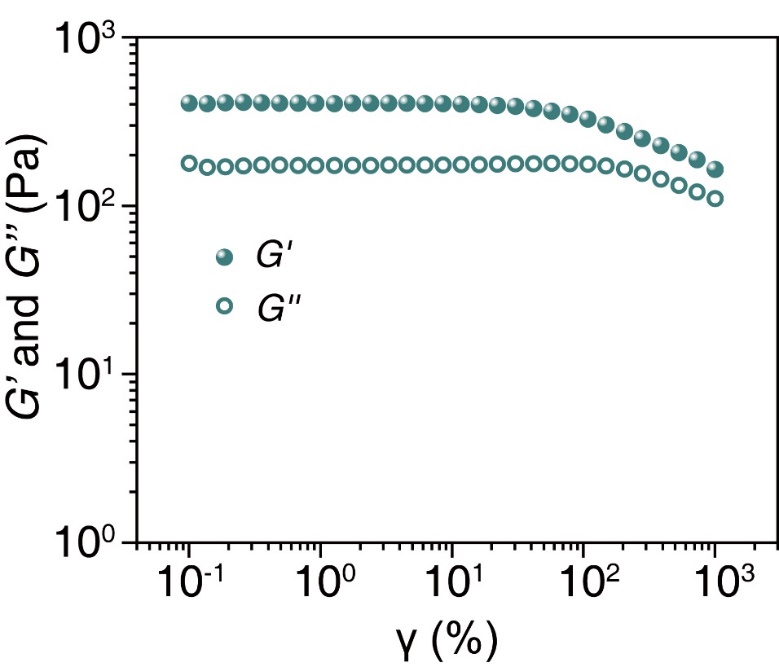


**Figure S5.** Rheological characteristics with the oscillation strain sweep of BAPC1.


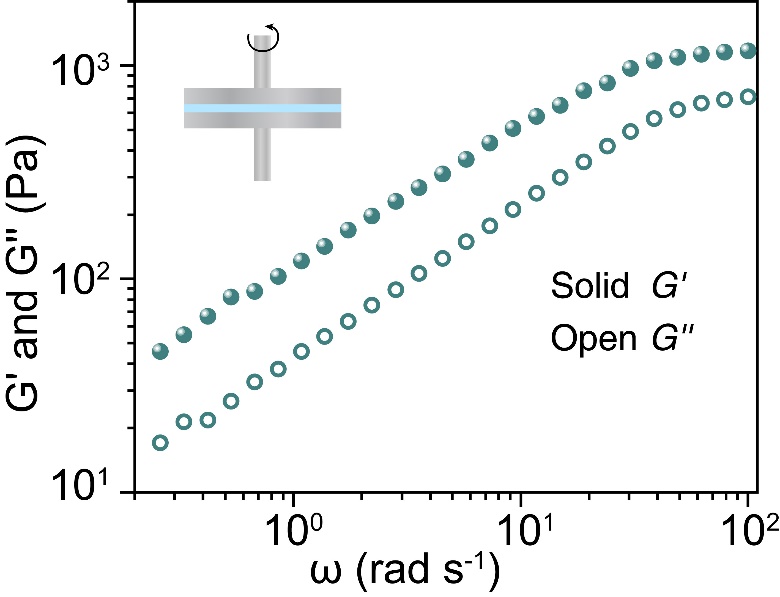


**Figure S6.** Rheological characteristics with the frequency scanning of BAPC1.


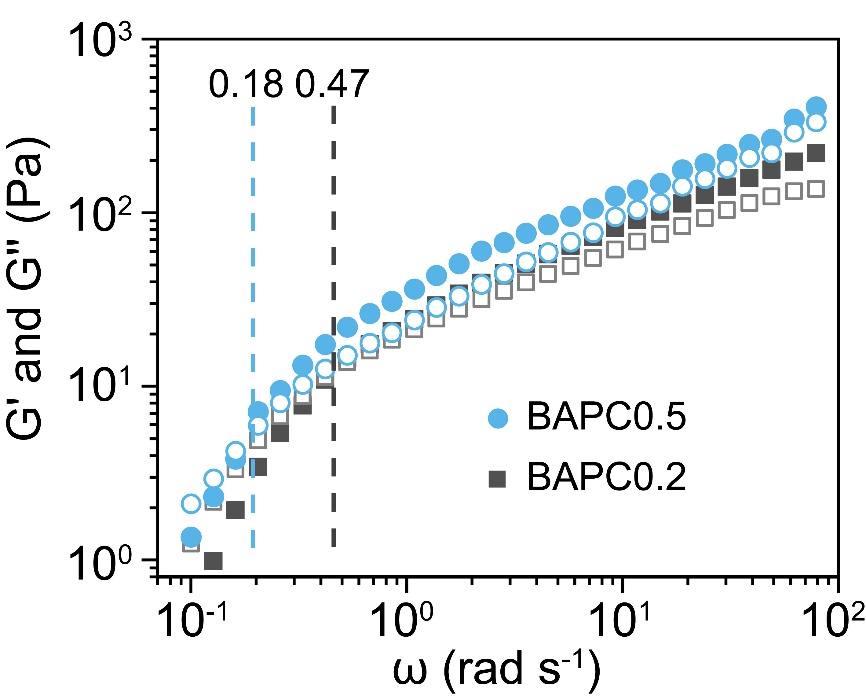


**Figure S7.** Rheological characteristics with the frequency scanning of weakly crosslinked BAPC0.2 and BAPC0.5.


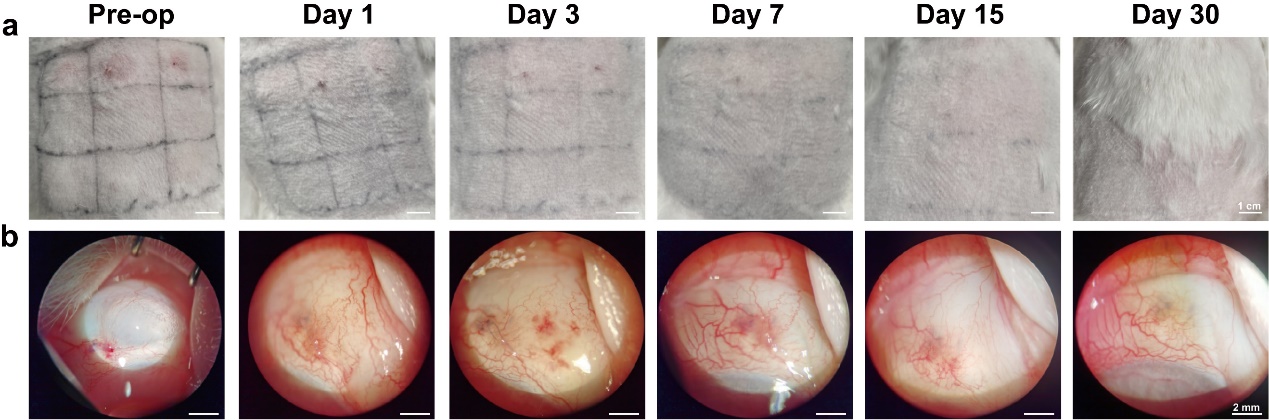


**Figure S8.** Subcutaneous and subconjunctival injection safety testing. (a) P(SBMA-co-AANa) hydrogel group (the first line), sham-puncture group (the second line), and normal saline control group (the last line) were observed on days 0, 1, 3, 7, 15, and 30 (scale bar = 1 cm), showed normal skin coloration and hair growth, without skin redness, swelling or induration. (b) No conjunctival hyperemia or edema was observed after subconjunctival gel injection (scale bar = 2 mm).


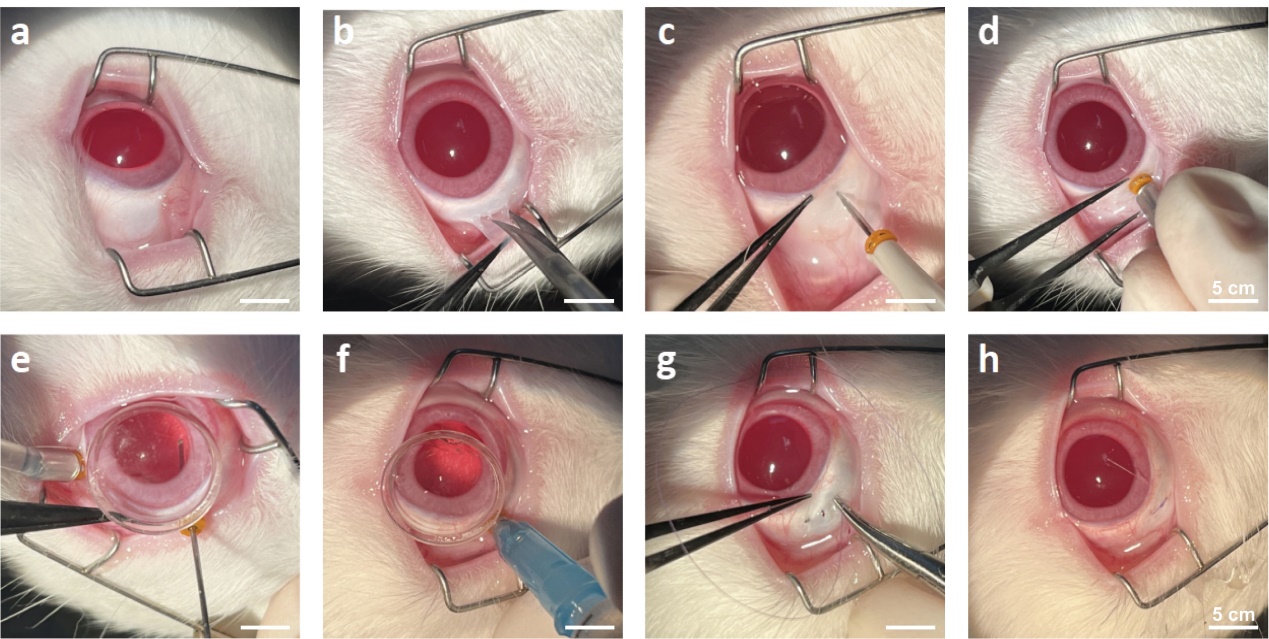


**Figure S9.** The procedure of vitrectomy and hydrogel injection. (a) The surgical eye was fully exposed, cleaned, disinfected, and dilated. Then, (b) dissecting the conjunctiva, (c) making the surgical incision, (d) fixing perfusion, (e) removing the vitreous, (f) injecting the P(SBMA-co-AANa) hydrogel into the vitrectomized eye, (g) sewing up the notch, and (h) dripping the antibiotic eye drops (scale bar = 5 cm).


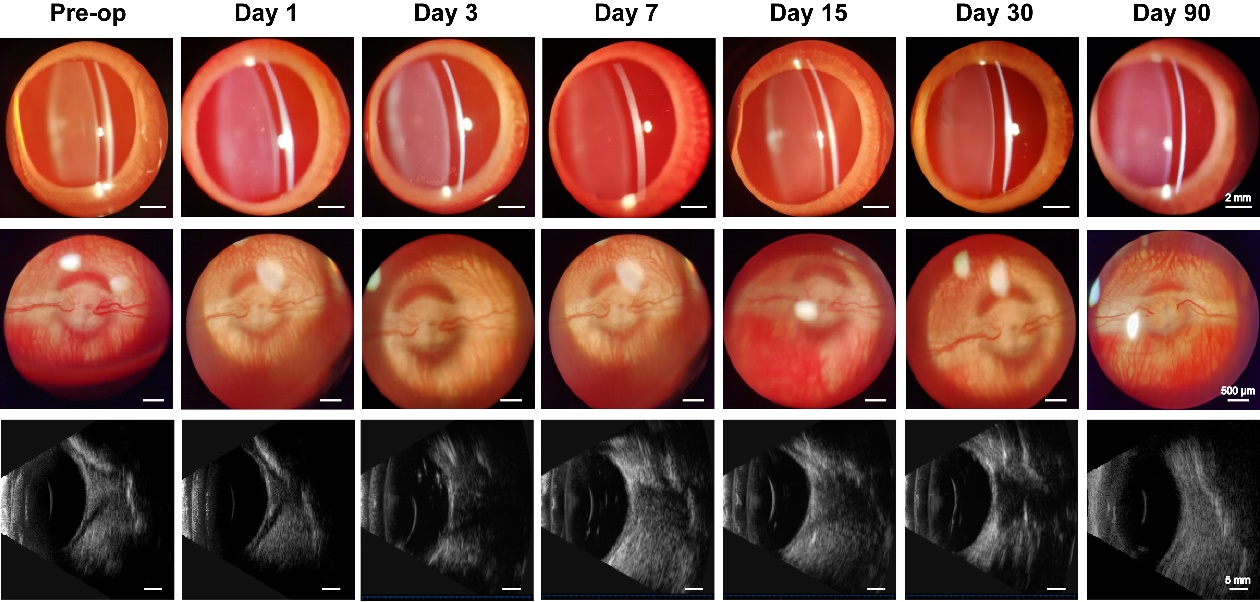


**Figure S10.** The anterior segment, fundus, and B-ultrasound results of the BSS group.


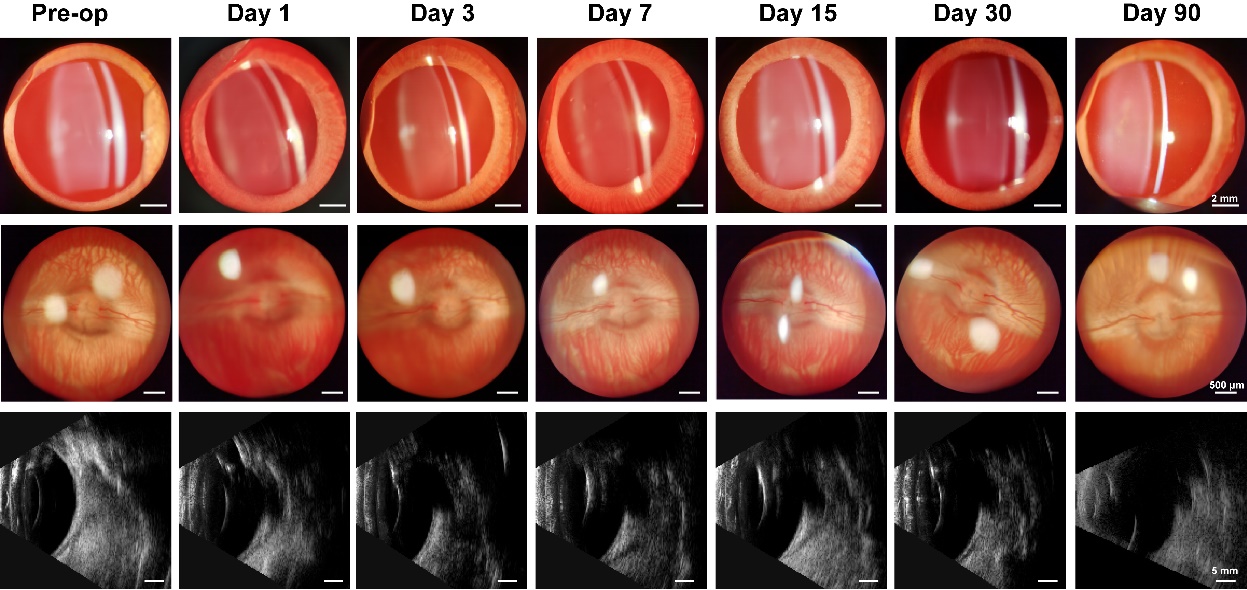


**Figure S11.** The anterior segment, fundus, and B-ultrasound results of the SO group.


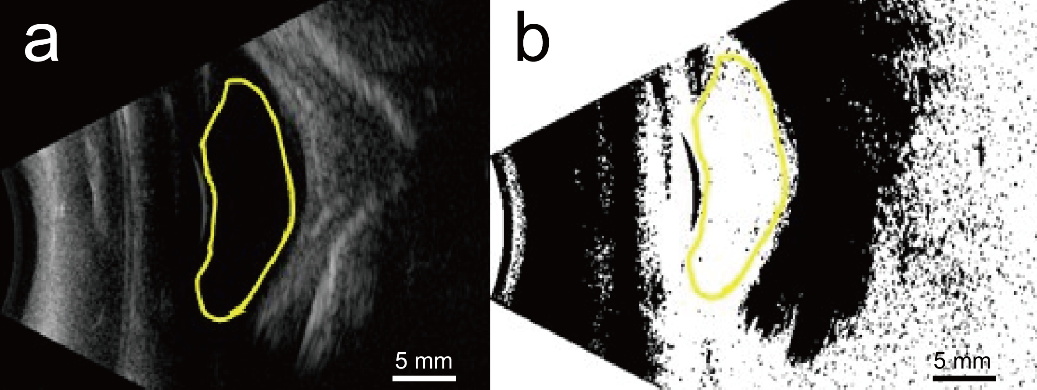


**Figure S****12.** Measuring method of B-ultrasound gray value of vitreous cavity. (a) Selecting the vitreous cavity as the region of interest. (b) Binarizing the image data, and measuring the black pixels on the region of interest (scale bar = 5 mm).


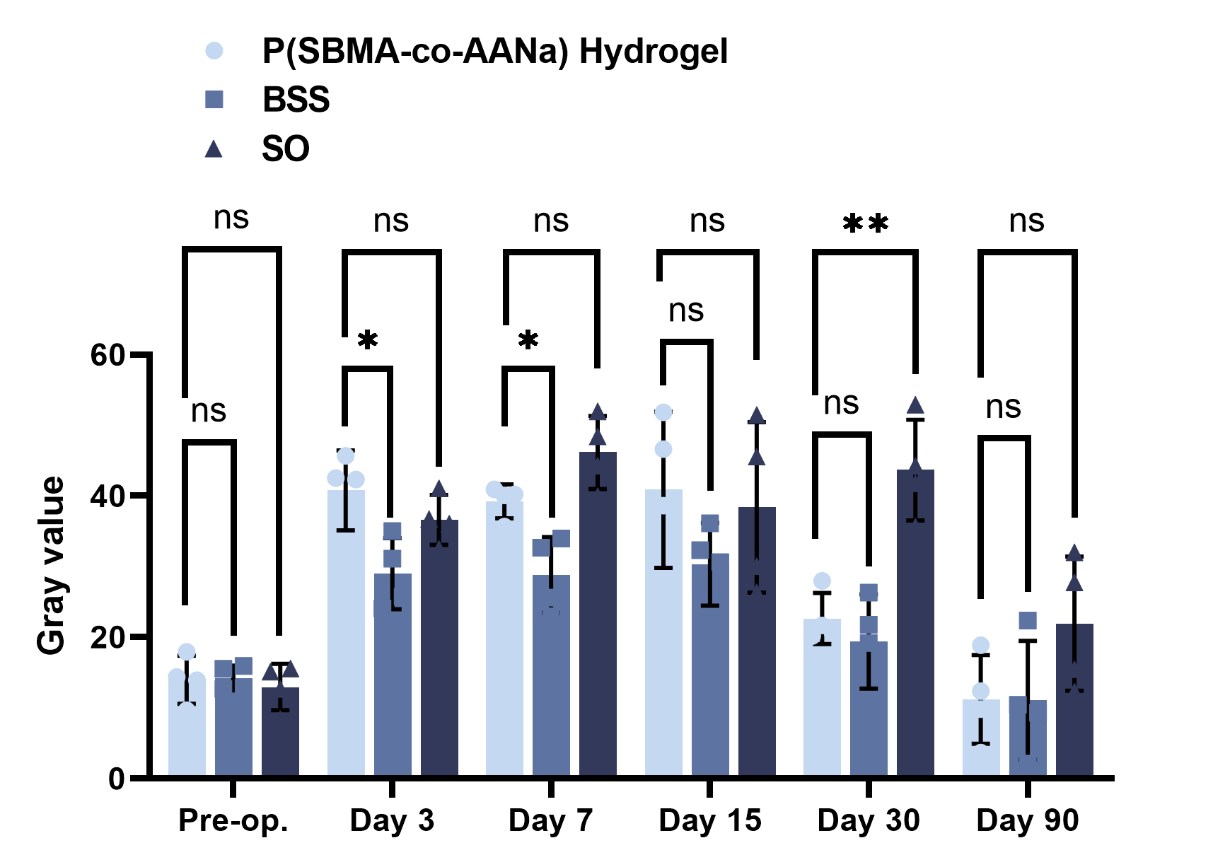


**Figure S13.** Measuring results of vitreous cavity gray value, indicating no significant difference between the P(SBMA-co-AANa) hydrogel and BSS groups. The gray value was presented as mean ± SD, and calculated by one-way ANOVA with Dunnett’s post hoc test, n = 4. *P < 0.05, ***P < 0.001, ****P < 0.0001.


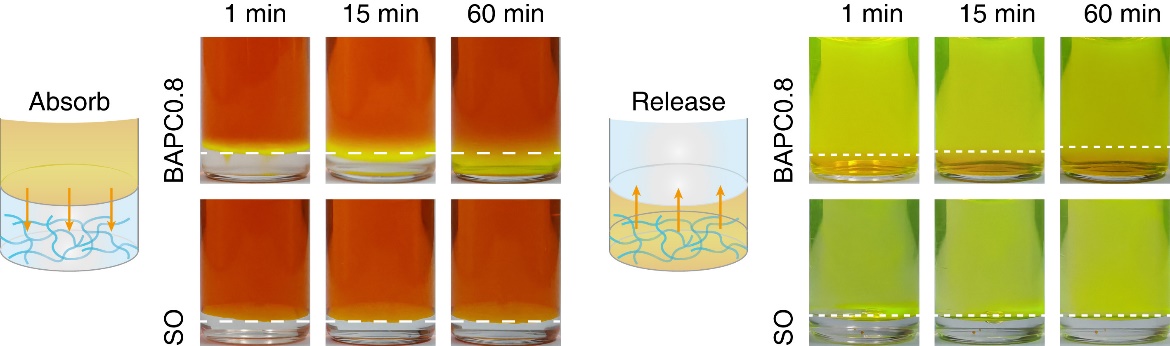


**Figure S14.** Evaluation of the absorption (Left) and release (Right) of fluorescein sodium dye by BAPC0.8 P(SBMA-co-AANa) hydrogel and SO.


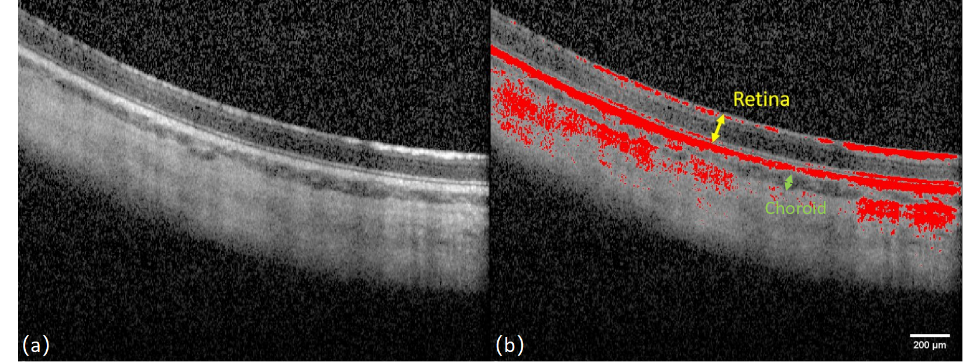


**Figure S15.** Measuring method of retinal and choroidal thickness. (a) The images were converted to 8-bit, and annotated with scale bar. (b) The threshold was adjusted for better retinal and choroidal identification (scale bar = 200 μm).


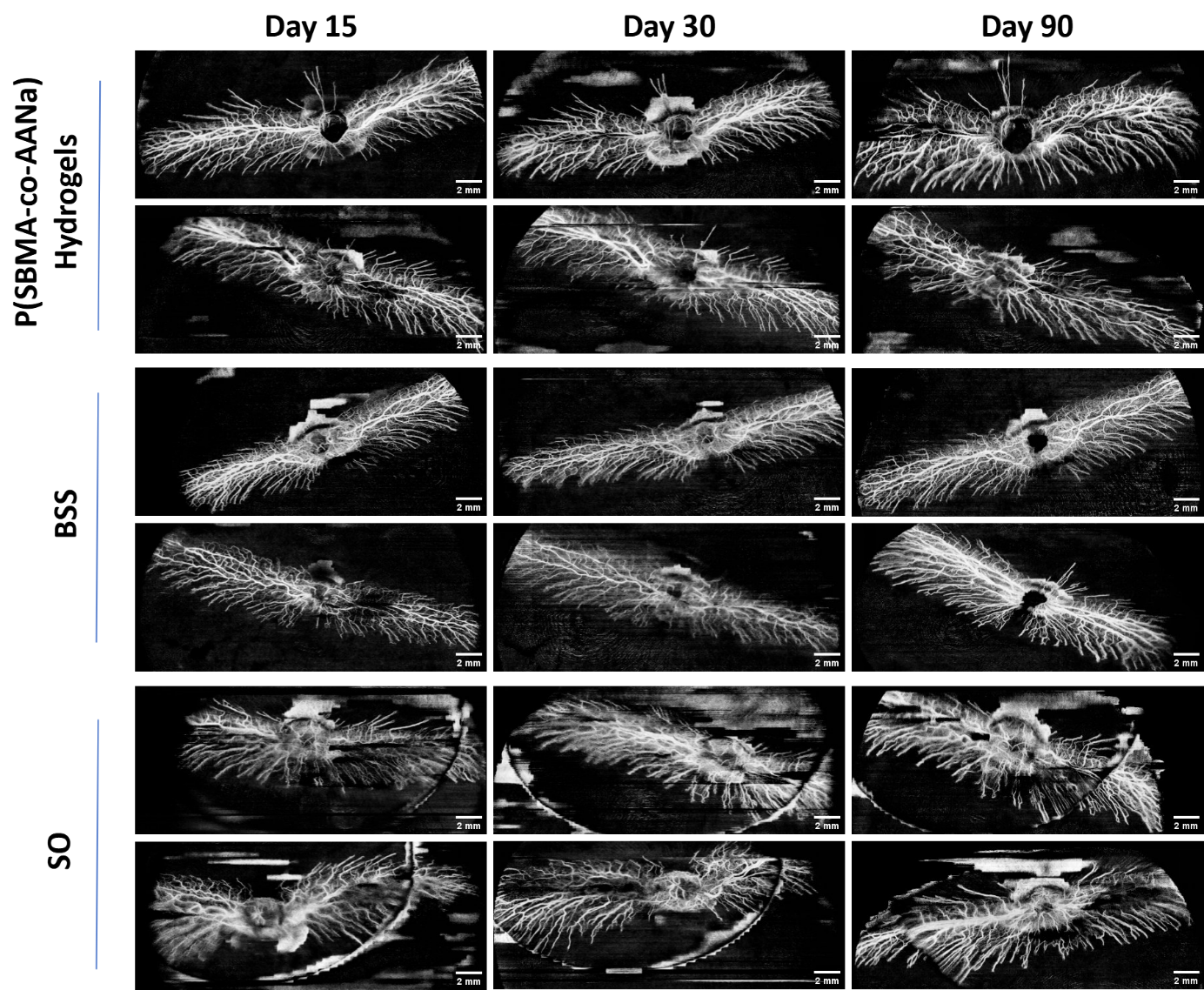


**Figure S16.** The OCTA results for the P(SBMA-co-AANa) hydrogel, BSS, and SO groups on days 15, 30 and 90 showed no obvious abnormalities in microcirculation (scale bar = 2 mm).


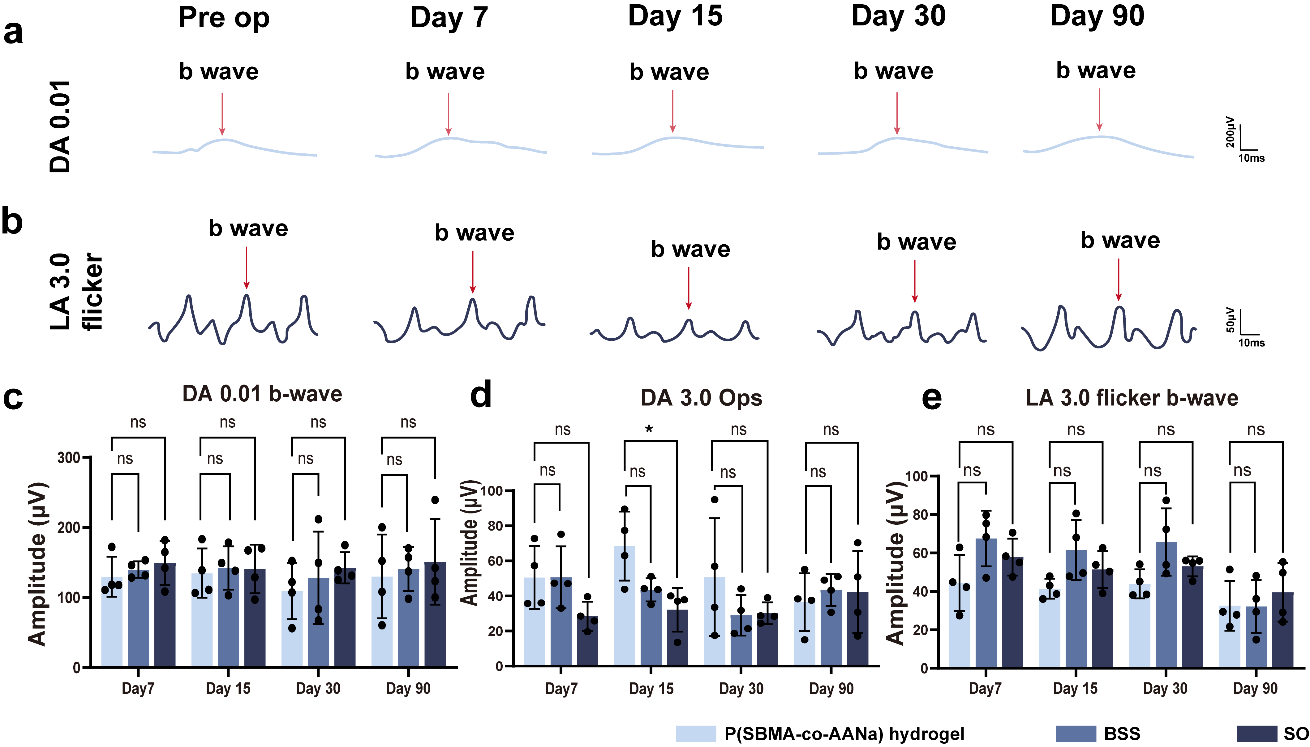


**Figure S17.** Results of (**a** and **c**) DA 0.01, (**b** and **e**) LA 3.0 flicker, and (**d**) DA 3.0 Ops in P(SBMA-co-AANa) hydrogel group. The ERG wave amplitude data were presented as mean ± SD and analyzed using one-way ANOVA with Dunnett’s post hoc test. Since the DA 0.01 b-wave amplitude data on day 30 deviated from a normal distribution, the Kruskal-Wallis test with Dunn's multiple comparisons test was employed for analysis. *P < 0.05, **P < 0.01; n = 4.


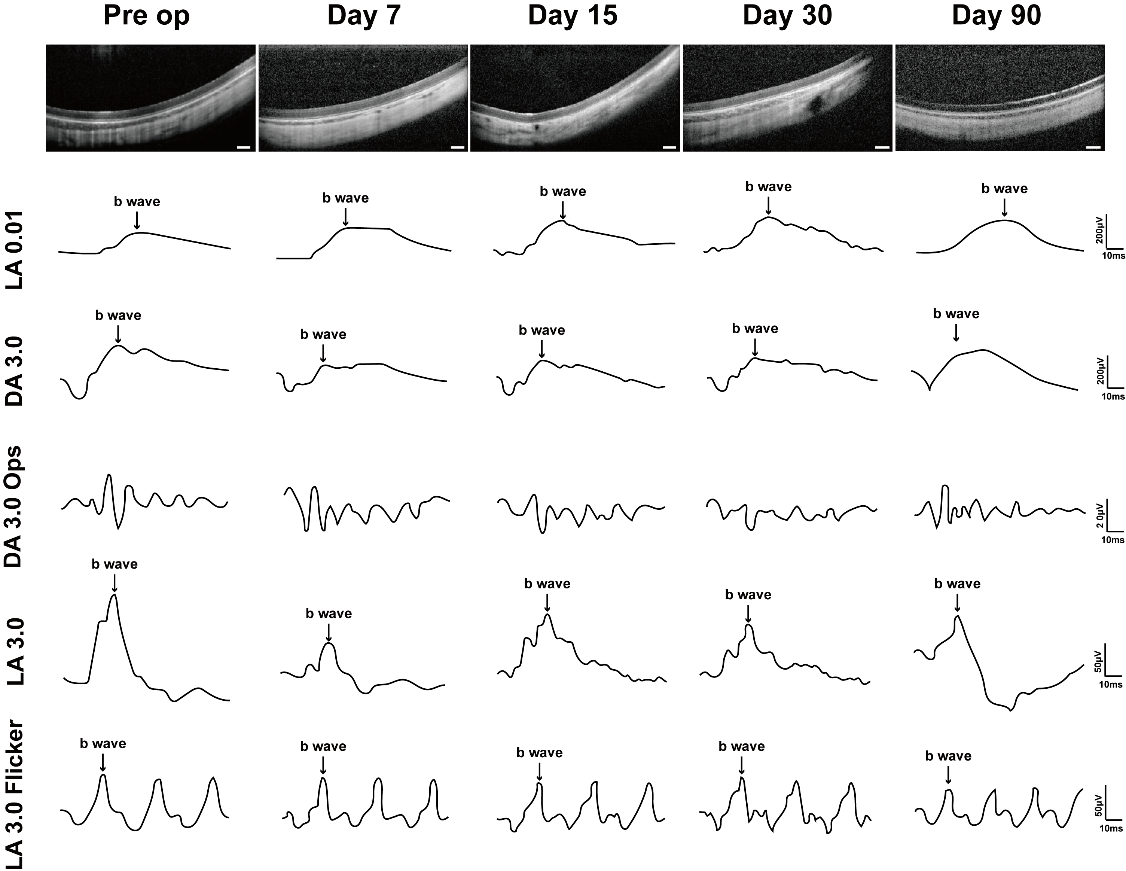


**Figure S18.** The OCT and ERG results of the BSS group (scale bar = 200 μm).


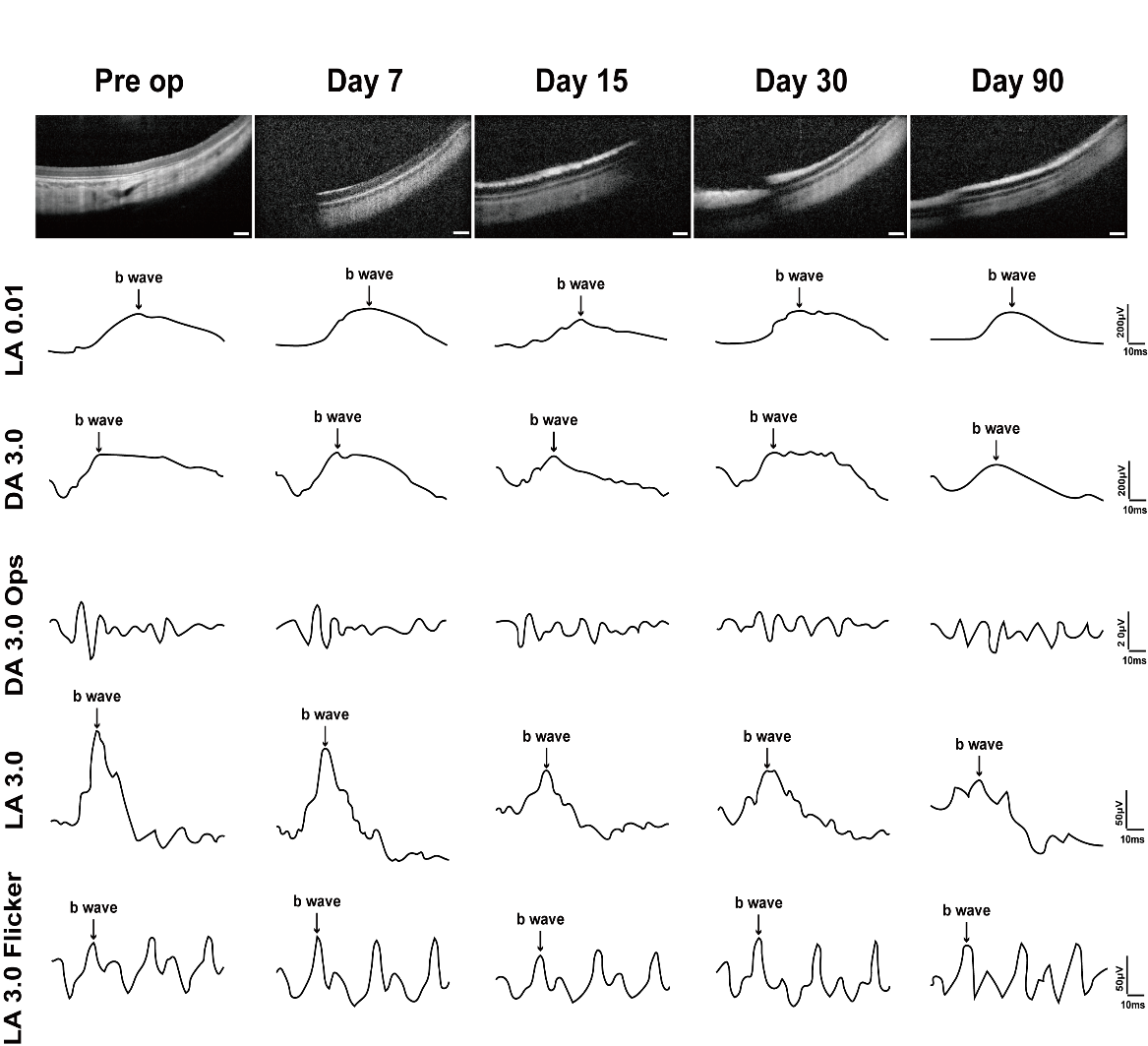


**Figure S19.** The OCT and ERG results of the SO group (scale bar = 200 μm).


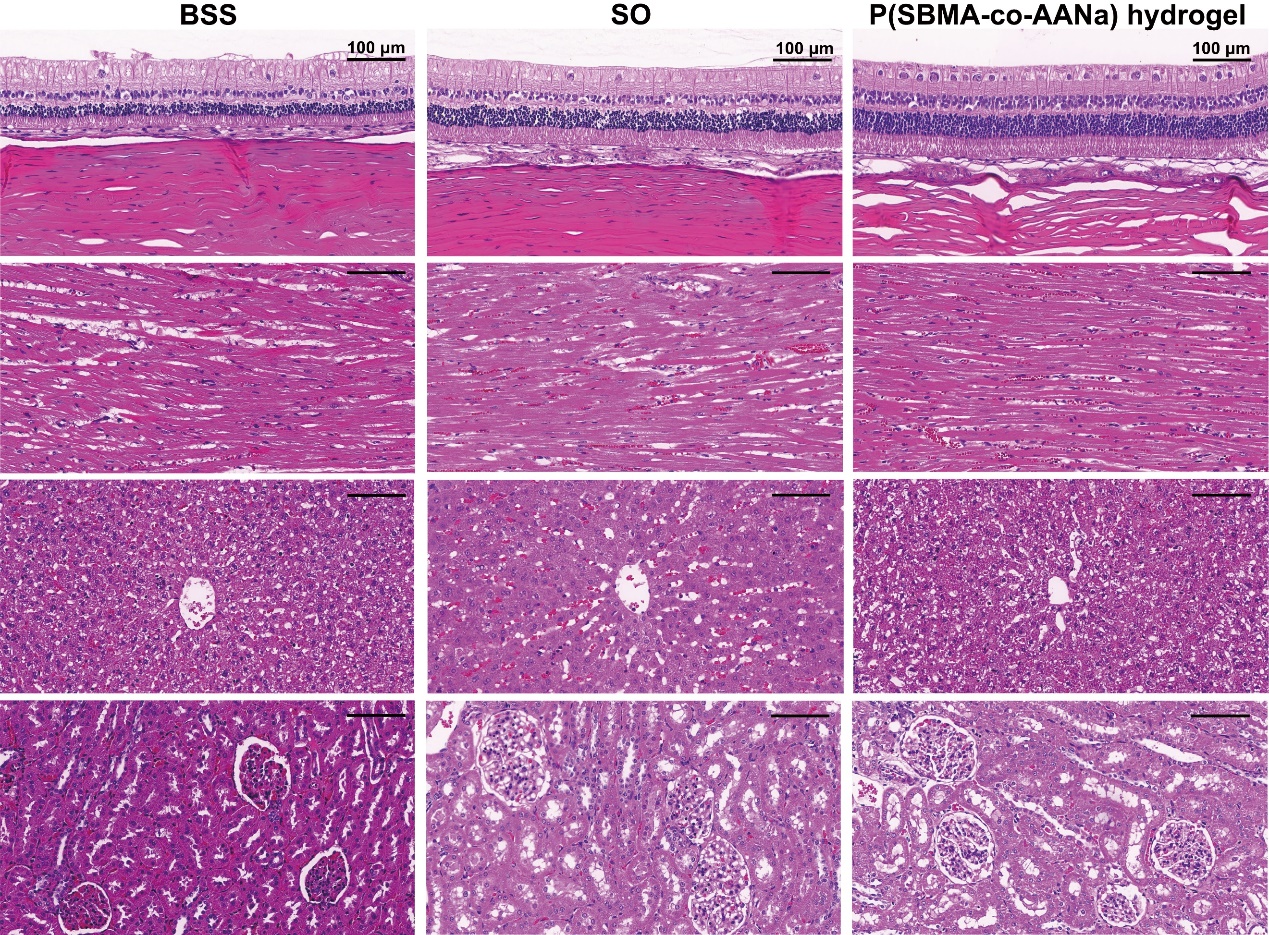


**Figure S20.** HE staining results of the retina, heart, liver, and kidney on the 30^th^ day after surgery (scale bar = 100 μm).


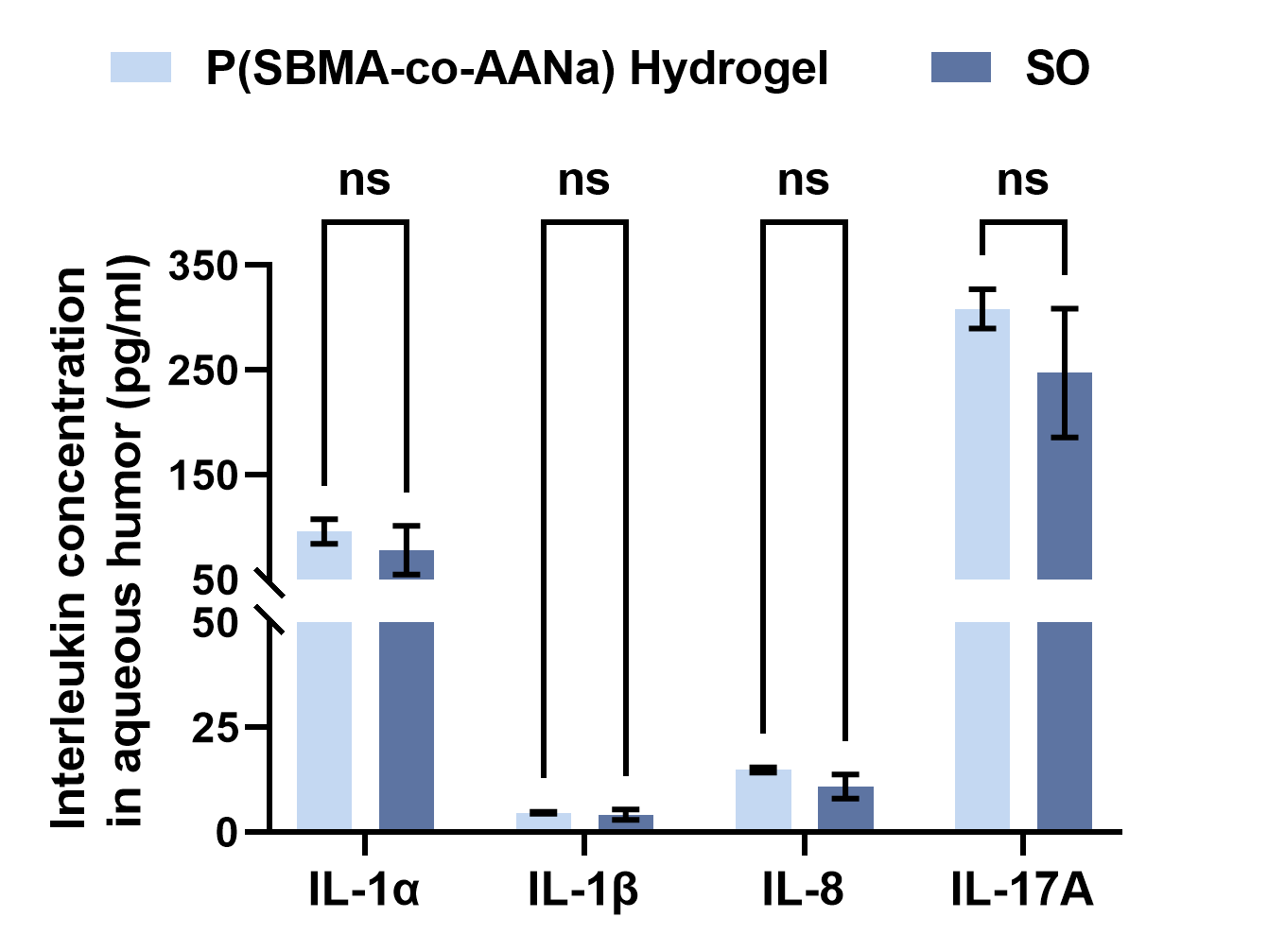


**Figure S21.** The interleukin concentration in aqueous humor on day 30. The data were presented as means ± SD, and analyzed by unpaired t-test, n = 4.


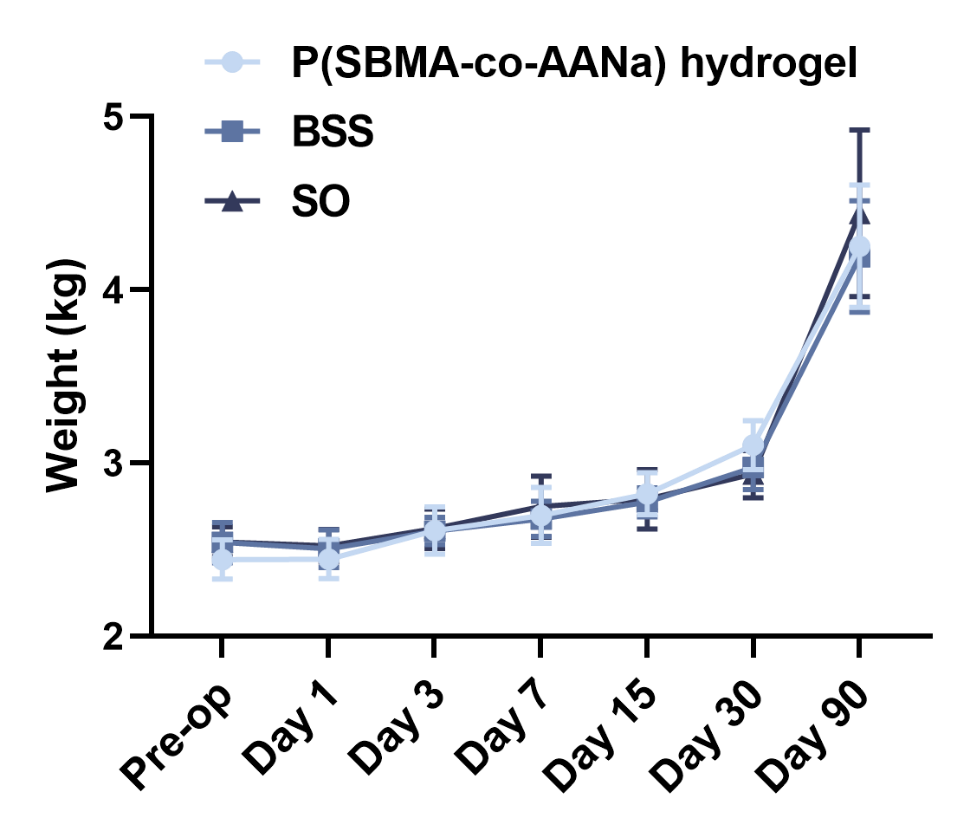


**Figure S22.** The weight gain curves of these three groups showed no statistically significant difference. The data were presented as means ± SD, and analyzed by one-way ANOVA with Dunnett’s post hoc test, n = 4.

**Materials and methods**

**Materials****.** Acrylic acid (AA), potassium peroxydisulfate (KPS), poly (ethylene glycol) diacrylate, tetramethylethylenediamine (TEMED), sodium hydrogen phosphate, and sodium dihydrogen phosphate were obtained from Aladdin Co. Ltd. Sulfobetaine methacrylate (SBMA) was purchased from Sigma-Aldrich Co. Ltd. Deionized water (18.2 MΩ at 25 ℃), obtained from the Waterpool ultrapure water purification system (WP-UP-YJ-20), was used in all experiments. All reagents used were of analytical grade.

**Preparation of hydrogels.** The hydrogel was prepared via in-situ copolymerization of AA with SBMA in an aqueous solution containing a trace amount of chemical crosslinker (PEGDA-1000). First, 1.392 g of SBMA was dissolved in 4 mL of deionized water. Next, 0.34 mL of AA and 0.1 mL of 2 wt% PEGDA-1000 solution were added, and the solution was continuously stirred to form a transparent mixture. The total monomer concentration (SBMA and AA) was 2 mol L^-1^. After 10 minutes, 1.08 ml of initiator (2 wt% KPS solution) and 0.03 ml of accelerator (TEMED) were added. After stirring for 5 minutes, the hydrogel precursor was formed. The precursor was then polymerized at 60 ℃ for 12 hours to generate hydrogels. The resulting hydrogels were neutralized to pH 7.4 using a phosphate buffer solution (PBS) and used in all subsequent experiments. Based on the different content of the chemical crosslinker, P(SBMA-co-AANa) hydrogels are defined as BAPCx, where x represents the mass concentration (wt ‰) of the chemical crosslinker relative to the monomer. Additionally, BAPC0 represented the polymers without any addition of the chemical crosslinker.

**Characterizations.** The hydrogels were washed with deionized water for several days and air-dried before fourier-transform infrared (FT-IR) analysis. FT-IR spectra were obtained using a Bruker Vertex FT-IR spectrometer, with a wavenumber range of 500-4000 cm^-1^. The morphology of hydrogel was observed using a scanning electron microscope (JSM-7900F, JEOL) with an accelerating voltage of 20 kV, following freeze-drying. The refractive indexes of hydrogels were measured with an Abbe refractometer (WYA-2W, Lumsail Industrial Inc., Shanghai, China). The visible light transmittance was measured with a UV-Vis spectrophotometer (UV 2450, Shimadzu, Japan) over the wavelength range of 350-800 nm at 37℃, with deionized water as the blank.

**Rheological measurement.** Rheological testing was performed on hydrogel samples neutralized with PBS and extruded through a small needle. Rheological properties were measured using an Anton Paar rheometer (MCR 92) with 15 mm parallel plates. An oscillation strain sweep was conducted with a strain range of 0.1-100% and angular frequency of 6.28 rad s^-1^ at 37°C. A frequency sweep was performed with angular frequencies ranging from 0.05-100 rad s^-1^ and a fixed strain of 0.5% at 37°C. Shear viscosity was measured by increasing the shear rate from 0.1 to 100 s^-1^ at 37°C. A time sweep was conducted with low and high oscillatory strains, switching between γ = 0.5% and γ = 200%, with a 2-minute interval for each strain.

**Permeability evaluation of betaine-based hydrogel.** 200 μL of hydrogel and silicone oil were evenly spread on the bottom of glass vials. After standing for 3 hours, 1 mL of fluorescein sodium solution diluted with PBS was added to each vial. The adsorption performance of the hydrogel and silicone oil for the dye was recorded through photographs at 1 minute, 15 minutes, and 60 minutes. To evaluate the permeability of the hydrogel, the fluorescein sodium solution was allowed to remain in contact with the hydrogel for 24 hours, after which the solution was replaced with 1 mL of fresh PBS solution. Subsequently, the release of fluorescein sodium solution from the hydrogel and silicone oil was recorded through photographs at intervals of 1, 5, 10, 15, 30, and 60 minutes.

**Cytotoxicity tests.** The cytotoxicity of P(SBMA-co-AANa) hydrogel was evaluated in the ARPE-19 cell line. ARPE-19 cells were seeded into 96-well plates at a seeding density of 2×10^4^ cells per well. After culturing for 24 hours, the cells were serum-starved in DMEM medium overnight. Then, the basal culture medium was removed, and various concentrations of P(SBMA-co-AANa) hydrogels (0%-10%) were added to each well. After the treatment period, the cell viability was assessed by adding CCK8 reagent, incubating the plates for 2 hours, and measuring absorbance at 450 nm. Cell viability (%) = (Optical density (OD) value of the sample − OD value of the blank / OD value of the control − OD value of the blank) ×100%.

**Live/Dead cell staining.** The ARPE-19 cells were seeded into 24-well plates at a density of 1×10^5^ cells per well. After culturing for 24 hours, the cells were serum-starved overnight in DMEM medium. Then, the basal culture medium was removed, and various concentrations of P(SBMA-co-AANa) hydrogels (0%-10%) were added to each well. After the indicated treatment, live and dead cells were stained with Calcein AM and PI, respectively, and the results were photographed by a fluorescence microscope (Leica DM4000B).

**In vitro wound-healing assay.** ARPE-19 cells were seeded into 24-well plates at a density of 8×10^4^ cells per well. Once the cells successfully adhered and reached 100% confluence, a 10-μL pipette tip was used to create straight scratches on the surface of wells. After washing off the non-adherent cells, culture medium containing different concentrations (0%, 1%, 5%, 10%) of BAPC0.8 was added to the wells, and the cells were further cultured. Images were captured at 0, 24, and 48 hours to observe the effect of the gel on ARPE-19 cell migration. Migration areas were analyzed using ImageJ 1.46r software.

**In vitro anti-cell adhesion test.** The P(SBMA-co-AANa) hydrogel and sodium hyaluronate (Pe-Ha-Luron® F, ALBOMED® GmbH) were applied to the bottom of 24-well plates. After incubating overnight, ARPE-19 cells were seeded into the blank group, control group (sodium hyaluronate), and experimental group (P(SBMA-co-AANa) hydrogel) at a density of 8×10^4^ cells per well. The culture was maintained for 24 h, followed by gentle washing of the plates three times with PBS. The remaining cells were stained by Calcein AM and PI, and cell adherence in all groups was observed under a fluorescence microscope (Leica DM4000B).

**In vitro anti-protein adhesion test.** The hydrogel disks, equilibrated in advance, were immersed in 1 mL of BSA solution (2 mg mL^−1^) at 37℃ for 90 min. After incubation, the disks were washed with PBS three times to remove the loosely adsorbed proteins. Then the adsorbed proteins were detached by ultrasound. The concentration of the adsorbed proteins was determined using the micro-BCA protein assay, following the provided protocol. Protein quantities were calculated based on the concentration of the standard protein solution. All tests were performed in triplicate. For comparison, nonspecific protein adsorption by silicon-based elastomers was evaluated in the same manner.

**Animals.** Eighteen adult male New Zealand White rabbits (weighing 2.5-3.0 kg; Taiping Biological Technology Co. Ltd., Hunan, China) were included in our study. All procedures and treatments adhered to the guidelines set by the government committee on animal experimentation at the 2nd Xiangya Hospital of Central South University. Ethical approval was granted by the Institutional Animal Care and Use Committee at the 2nd Xiangya Hospital of Central South University (Approval No.2020590).

**Vitrectomy surgery.** After general anesthesia was induced with a combination of intramuscular Chlorpromazine (30 mg kg^-1^) and 3% intravenous Pentobarbital sodium (1 mg kg^-1^), compound tropicamide eye drops (containing 5 mg tropicamide and 5 mg phenylephrine in 1 mL) were used to dilate the pupils. Standard 3-port, 23-gauge pars-plana vitrectomy was performed on both eyes of rabbits, with sclerotomies 3.0 mm from the limbus, to remove the vitreous. P(SBMA-co-AANa) hydrogel, BSS, and SO were injected into the eye (1.0-1.5 mL per eye). Gatifloxacin Eye Ointment, Tobramycin-Dexamethasone Eye Ointment (0.3% Tobramycin and 0.1% Dexamethasone), and 1% Prednisolone Acetate eye drops were applied for 14 days for post-surgical inflammation and infection prophylaxis. All surgical procedures were performed by the same experienced surgeon. One eye was excluded due to bleeding caused by accidental contact with the retina during surgery, and another eye was excluded due to cataract formation accelerated by contact with the lens during the procedure.

**Weight measurement.** The weight was measured using a digital weighing scale (DY-1, Shanghai Guang Zheng Medical Instrument Co., Ltd) on days 0 (pre-operation), 1, 3, 7, 15, 30, and 90.

**Intraocular pressure (IOP) measurements.** The IOP was measured at the center of the cornea using a TonoVet tonometer (Icare, Finland) on both eyes of the rabbits on days 0 (pre-operation) 1, 3, 7, 15, 30, and 90.

**In vivo ophthalmic imaging.** The morphology of the natural gel was observed non-invasively using a slit-lamp. The anterior and posterior segments photography was taken on days 0 (pre-operation), 1, 3, 7, 15, 30, and 90. The fundus was examined and photographed using a 90 Diopter non-contact slit-lamp lens (Volk Optical Inc.) at each time point. All slit-lamp assessments were performed and recorded by an experienced ophthalmologist.

**In vivo optical coherence tomography.** Retinal and choroidal layers were examined in detail using real-time spectral-domain optical coherence tomography (Heidelberg Engineering). Each animal underwent repeated SD-OCT on days 0 (pre-operation), 1, 7, 15, 30, and 90. Longitudinal and transverse line scans were performed at the posterior pole (1-2 optic disc diameters away from the optic disc) using the 30° visual field acquisition mode. Using ImageJ 1.46r software for retinal and choroidal layer thickness analysis (**Figure S15**). And the retinal vascular perfusion area was examined using optical coherence tomography angiography system (Dream OCT, Intalight Science and Technology Ltd., Shanghai, China) on days 15, 30 and 90 after surgery.

**In vivo follow-up by B- ultrasonography.** The turbidity degree of the vitreous cavity was examined by a B-ultrasonography (AVISO). Each animal was checked on days 0 (pre-operation), 3, 7, 15, 30, and 90. The opacity of the vitreous cavity was analyzed using ImageJ 1.46r software (**Figure S12**).

**Retinal function assessments by** **electroretinogram (ERG).** To estimate the function of retinal cells, repeated ERG testing was performed. All the rabbits were dark-adapted for 30 minutes before undergoing full-field ERG, and their pupils were dilated as previously described. ERGs were recorded using MonPackONE electrophysiological equipment (METRO VISION) under anesthesia. Full-field ERG protocols for rabbits followed those recommended for human patients by the International Society for Clinical Electrophysiology of Vision (ISCEV). The components of the ERG examination are named according to the eye's adaptive state (dark-adapted [DA] or light-adapted [LA]) and the stimulus strength in candela-seconds per square meter. Thus, DA 0.1 refers to a 0.1 cd.s.m^-2^ flash delivered under dark adaptation, and LA 3.0 refers to a 3.0 cd.s.m^-2^ flash delivered under photopic conditions.

**Histopathological examination.** The rabbits were euthanized, and their eyeballs were enucleated. The entire globes were immersed in FAS specimen fixative (Servicebio, Wuhan) overnight. After removing the anterior segments, full-thickness samples (6×6mm, retina→sclera) were excised and embedded in paraffin (n=5 slides per eye, one sample was taken from the posterior pole, corresponding to the region scanned by OCT). Sections were cut to 5 µm using a microtome (Leica RM2016) and stained with H&E. Additionally, the heart, liver, and kidney were dissected and subjected to H&E staining.

**Inflammatory cytokine detection.** On postoperative day 30, a 1 mL syringe was used to puncture the corneal limbus, and approximately 100 µL of aqueous humor was extracted for Quantibody® Rabbit Cytokine Array 1 testing.

**Statistical methods.** ImageJ was utilized to process and calculate the scratch area, the number of adhered cells, the B-ultrasound gray value, and the retinal and choroidal thickness. The quantitative data were presented as mean ± SD. The Shapiro-Wilk test was used to assess the normality of the data, and the Brown-Forsythe test was used to assess the homogeneity of variances. An unpaired t-test was employed to analyze protein adsorption of the P(SBMA-co-AANa) hydrogel and interleukin concentration in aqueous humor. One-way ANOVA with Dunnett’s post hoc test was used to analyze data for cell viability, cell counting, the 48-hour migration area of ARPE-19 cells, intraocular pressure, retinal and choroidal thickness, B-mode ultrasound gray value, ERG wave amplitude, and weight. For the 24-hour migration area of ARPE-19 cells, DA 3.0 Ops amplitude data on day 7, LA 3.0 b-wave amplitude data on day 30, and DA 0.01 b-wave amplitude data on day 30, which did not follow a normal distribution, the Kruskal-Wallis test with Dunn’s multiple comparisons test was applied. All statistical analyses were conducted using GraphPad Prism (v9.5.1, GraphPad Software Inc.) with a significance level set at 0.05.
